# HELLS Is Negatively Regulated by Wild-Type P53 in Liver Cancer by a Mechanism Involving P21 and FOXM1

**DOI:** 10.3390/cancers14020459

**Published:** 2022-01-17

**Authors:** Stefanie Schuller, Jan Sieker, Philip Riemenschneider, Bianca Köhler, Elisabeth Drucker, Sofia M. E. Weiler, Daniel Dauch, Carsten Sticht, Benjamin Goeppert, Stephanie Roessler, Silvia Ribback, Kai Breuhahn, Falko Fend, Frank Dombrowski, Kerstin Singer, Stephan Singer

**Affiliations:** 1 Institute of Pathology, University Medicine Greifswald, 17475 Greifswald, Germany; stefanie.schuller@uni-greifswald.de (S.S.); js132977@uni-greifswald.de (J.S.); philip.riemenschneider@med.uni-tuebingen.de (P.R.); Bianca.Koehler@med.uni-tuebingen.de (B.K.); lisi.drucker@gmail.com (E.D.); silvia.ribback@uni-greifswald.de (S.R.); frank.dombrowski@uni-greifswald.de (F.D.); Kerstin-Anna.Singer@uni-tuebingen.de (K.S.); 2 Institute of Pathology, University Hospital Tuebingen, 72076 Tuebingen, Germany; Falko.Fend@med.uni-tuebingen.de; 3 Institute of Pathology, University Hospital Heidelberg, 69120 Heidelberg, Germany; sofia.weiler@med.uni-heidelberg.de (S.M.E.W.); benjamin.goeppert@med.uni-heidelberg.de (B.G.); Stephanie.Roessler@med.uni-heidelberg.de (S.R.); kai.breuhahn@med.uni-heidelberg.de (K.B.); 4 Department of Internal Medicine VIII, University Hospital Tuebingen, 72076 Tuebingen, Germany; daniel.dauch@uni-tuebingen.de; 5 Translational Gastrointestinal Oncology Group, German Consortium for Translational Cancer Research (DKTK), German Cancer Research Center (DKFZ), 69120 Heidelberg, Germany; 6 Core Facility Platform Mannheim (CFPM), NGS Core Facility, Medical Faculty Mannheim, Heidelberg University, 68167 Mannheim, Germany; carsten.sticht@medma.uni-heidelberg.de; 7 Cluster of Excellence iFIT (EXC 2180) “Image-Guided and Functionally Instructed Tumor Therapies”, University of Tuebingen, 72076 Tuebingen, Germany

**Keywords:** chromatin remodeling, HCC, gene repression, P53 network

## Abstract

**Simple Summary:**

The tumor suppressor protein P53 is a major player in preventing liver cancer development and progression. In this study we could show that P53 negatively regulates the expression of Helicase, lymphoid specific (*HELLS*), previously described as an important pro-tumorigenic epigenetic regulator in hepatocarcinogenesis. The regulatory mechanism included induction of the P53 target gene P21 (*CDKN1A*) resulting in repression of *HELLS* via downregulation of the transcription factor Forkhead Box Protein M1 (FOXM1). Our in vitro and in vivo findings indicate an important additional aspect of the tumor suppressive function of P53 in liver cancer linked to epigenetic regulation.

**Abstract:**

The major tumor suppressor P53 (*TP53*) acts primarily as a transcription factor by activating or repressing subsets of its numerous target genes, resulting in different cellular outcomes (e.g., cell cycle arrest, apoptosis and senescence). P53-dependent gene regulation is linked to several aspects of chromatin remodeling; however, regulation of chromatin-modifying enzymes by P53 is poorly understood in hepatocarcinogenesis. Herein, we identified Helicase, lymphoid specific (*HELLS*), a major epigenetic regulator in liver cancer, as a strong and selective P53 repression target within the SNF2-like helicase family. The underlying regulatory mechanism involved P53-dependent induction of P21 (*CDKN1A*), leading to repression of Forkhead Box Protein M1 (*FOXM1*) that in turn resulted in downregulation of *HELLS* expression. Supporting our in vitro data, we found higher expression of HELLS in murine HCCs arising in a *Trp53−/−* background compared to *Trp53+/+* HCCs as well as a strong and highly significant correlation between *HELLS* and *FOXM1* expression in different HCC patient cohorts. Our data suggest that functional or mutational inactivation of P53 substantially contributes to overexpression of HELLS in HCC patients and indicates a previously unstudied aspect of P53′s ability to suppress liver cancer formation.

## 1. Introduction

Primary liver cancer ranks third of all cancer-related deaths with one million incident cases globally in 2016 [1]. The most common histological subtype of primary liver cancer is hepatocellular carcinoma (HCC) characterized by an extremely poor prognosis [2] and limited therapeutic options [3].

Among essential players in (liver) cancer biology is the tumor suppressor protein P53, serving as a major barrier against malignant transformation and progression [4,5]. More than 50% of all human cancers and 10–50% of HCC (depending on the etiology/risk factors including HBV, HCV and aflatoxin exposure) harbor *TP53* mutations, highlighting the importance of P53 in (hepato-)carcinogenesis [4,6]. P53 responds to a variety of different cellular stresses (e.g., DNA damage, hypoxia, oncogene activation and telomere shortening) by activating or suppressing subsets of its numerous target genes to induce different cellular outcomes [4,5,7]. Mild stress levels lead to DNA repair or cell cycle arrest, allowing further survival of the cell, while extensive stress, like irreversible DNA damage, induces apoptosis or senescence [5,7,8]. Among P53 target genes the cyclin-dependent kinase inhibitor P21/*CDKN1A* represents one of the most important and best characterized direct P53 targets [4]. P21/*CDKN1A* is a key mediator of the cell cycle arrest response, but can also be involved in all other aforementioned P53 responses [4,9,10]. Moreover, P21 is critical for mediating indirect P53-dependent transcriptional repression associated with the repressive DREAM complex [11,12]. Indirect gene repression by P53 via the P21-DREAM axis has been described for over 200 genes [12] including SURVIVIN/*BIRC5*, *CDC25C* and *PLK1* [13].

There is a complex interplay between the P53 network and chromatin remodeling factors in different settings. For instance, histone acetyltransferases (HATs) such as p300/CBP, pCAF, GCN5 or TIP60 are recruited in a P53-dependent manner for histone acetylation within the proximity of P53 response elements (REs) to allow access of the general transcription machinery [5]. To fully activate transcription of P21/*CDKN1A* after DNA damage, the histone variant H2A.Z is recruited in a P53-dependent manner. In addition, to enable transcription of *GADD45* after UV irradiation PRMT1 and CARM1 interact via P53 with p300/CBP [14]. Furthermore, it could be shown that mutant P53 regulates chromatin architecture by mediating nucleosomal displacement by cooperating with the SWI/SNF chromatin remodeling complex [15].

The first SNF2/SWI2 enzyme was discovered in yeast and named after their properties uncovered in different test settings: Sucrose nonfermenting mutant (SNF2) and mating-type switching mutant (SWI2) [16,17]. The most commonly used name in the community is SNF2, which in turn is part of the helicase superfamily 2 (SF2). The SNF2 family consists of approximately 30 different enzymes in humans which are further subclassified into six groups based on the structure of their helicase domain: SNF2-like, SWR1-like, SSO1653-like, RAD54-like, RAD5/16-like, SMARCAL1 [18]. SNF2 enzymes have been shown to regulate DNA accessibility and affect DNA methylation, histone modification, histone variant exchange and higher order chromatin structure [18]. Recently, Law et al. identified the lymphoid-specific helicase (LSH, HELLS/*HELLS*), a member of the SNF2-like helicase protein family, to be highly upregulated in HCC [19]. HELLS modifies and regulates chromatin structure in eukaryotes, interacts with the DNA methyltransferase DNMT1 and alters DNA methylation patterns [18]. Law et al. could show that HELLS is a major epigenetic regulator in HCC which orchestrates nucleosome occupancy resulting in silencing of multiple (liver) cancer relevant genes (e.g., E-Cadherin/*CDH1*, *FBP1*, *IGFBP3*, *XAF1* and *CREB3L3*) [19], also by DNA-methylation-independent mechanisms. Furthermore, overexpression of *HELLS* correlated with a more aggressive HCC phenotype by promoting cell proliferation, migration and tumorigenicity in vivo and was observed particularly in *TP53* mutant HCCs [19]. However, a regulatory molecular mechanism between the P53 network and HELLS has not been explored in liver cancer so far.

The transcription factor Forkhead Box protein M1 (FOXM1) belongs to the Forkhead box (FOX) protein superfamily and is pivotal for the regulation of a great variety of cancer-relevant processes, such as cell differentiation, cell proliferation, cell cycle progression, DNA damage repair, apoptosis and angiogenesis [20]. Accordingly, FOXM1 is found overexpressed in numerous malignancies, including HCC [20]. Kalinichenko et al. [21] could show that FOXM1 is essential for tumor progression in HCC suggestive of a promising therapeutic target. In line with these findings, it was demonstrated by Park et al. [22] that FOXM1 is involved in epithelial–mesenchymal transition (EMT), cell motility and invasion and thereby is relevant for metastasis formation. In vivo studies by Gusarova et al. [23] could show that mice overexpressing FOXM1 developed particularly aggressive tumors following chemical induced hepatocarcinogenesis. Conversely, inhibition of FOXM1 function resulted in reduced tumor cell survival and increased apoptosis in vivo and in vitro. FOXM1 overexpression in HCC being correlated with tumor cell proliferation, genomic instability and microvessel density was linked to extracellular signal-regulated kinase (ERK) and glioblastoma-associated oncogene 1 (GLI1) by Calvisi et al. [24]. Furthermore Chen et al. [25] have shown that FOXM1 also binds a non-canonical cell cycle genes homology region (CHR) element, besides the canonical binding site common to forkhead (FOX) transcription factors.

Here, we show that HELLS represents a strong P53 repression target in liver cancer by a mechanism involving the cyclin-dependent kinase inhibitor P21/*CDKN1A* and the transcription factor Forkhead Box Protein M1 (FOXM1). Consequently, functional or mutational loss of P53′s wild type activity contributes to overexpression of *HELLS* in a significant fraction of HCCs and suggests a previously unrecognized aspect of P53′s ability to suppress liver cancer formation.

## 2. Material and Methods

### 2.1. Cell Culture and Drug Treatment

HepG2 and Sk-Hep1 cell lines were purchased from ATCC (American Type Culture Collection, Manassas, VA, USA) and HLE, HLF, HUH7 and HUH6 cell lines were purchased from JCRB (Japanese Collection of Research Bioresources Cell Bank, Kansas City, KS, USA). All aforementioned cell lines have been validated using STR-analyses (Leibniz-Institut, DSMZ, Braunschweig, Germany). Mycoplasma contamination has regularly been excluded by using the MycoAlert^TM^ Plus Mycoplasma Detection Kit (LT07, Lonza, Cologne, Germany). HepG2 cells were cultured in RPMI 1640 Medium (Sigma, Taufkirchen, Germany); Sk-Hep1, HLE, HLF, HUH7 and HUH6 cells were maintained in DMEM (Sigma, Taufkirchen, Germany) each supplemented with 10% fetal calf serum and 1% penicillin/streptomycin. Cells were incubated at 37 °C in an atmosphere containing 5% CO_2_. Nutlin-3a (N6287, Sigma, Taufkirchen, Germany) and Camptothecin (CPT) (Hölzel Diagnostika, Cologne, Germany) were dissolved in DMSO (A994.2, Carl Roth GmbH und Co. KG, Karlsruhe, Germany) and used at a final concentration of 10 µM (Nutlin-3a) and 300 nM (CPT) for 24 h or 48 h, as indicated.

### 2.2. Transfection of siRNA and Plasmid Constructs

The siRNA transfections were performed using Oligofecatmin (Invitrogen, Karlsruhe, Germany), according to the manufacturer’s instructions. Gene-specific siRNAs (see Table 1) were purchased from Qiagen (Hilden, Germany) and Eurofins (Eurofins Genomics Germany GmbH, Ebersberg, Germany), and used at a final concentration of 50 nM. The QIAGEN ALL-Stars duplex served as a negative control for all knockdown experiments.

The FOXM1 HA-N plasmid (pDEST26_HA-N) was kindly provided by the Breuhahn Laboratory (Institute of Pathology, University Hospital Heidelberg, Heidelberg, Germany). Transient transfections with the respective plasmid concentrations were performed using FuGENE HD Transfection Reagent (Promega, Madison, WI, USA) according to the manufacturer’s recommendations.

### 2.3. Gel Electrophoresis and Immunoblotting

Cell lysis was performed by using a cell lysis buffer (New England Biolabs, Frankfurt, Germany) supplemented with a protease inhibitor cocktail (complete Mini EDTA-free, Roche Diagnostics GmbH, Mannheim, Germany). The Bradford assay (#500-006, Protein Assay Dye Reagent Concentrate, Bio Rad Laboratories GmbH, Munich, Germany) was used to measure the protein content of whole cell lysates. Samples were heated to 90 °C for 5 min, proteins were separated by SDS/PAGE and transferred to nitrocellulose membranes (Amersham Protran 0.2 µm NC, GE Healthcare Life science, Pittsburgh, USA). The membranes where blocked for 1 h in 5% milk/TRIS-buffered saline with Tween-20 (TBST) (milk powder, Carl Roth GmbH und Co. KG, Karlsruhe, Germany) and incubated overnight with the indicated antibodies diluted in blocking solution: Anti-HA (C29F4) rabbit monoclonal (dilution 1:500, #3724S, Cell Signaling Technology, Frankfurt, Germany); anti-HELLS rabbit polyclonal (dilution 1:500, GTX54157, GeneTex, Eching, Germany); anti-P21 rabbit polyclonal (dilution 1:250, sc-397, Santa Cruz, Heidelberg, Germany); anti-P21 mouse monoclonal (dilution 1:200, sc-6246, Santa Cruz, Heidelberg, Germany); anti-P53 mouse monoclonal (dilution 1:200, sc-17846, Santa Cruz, Heidelberg, Germany); anti-FOXM1 rabbit polyclonal (dilution 1:50, sc-500, Santa Cruz, Heidelberg, Germany). The detected ß-ACTIN (monoclonal mouse anti-actin antibody in a dilution 1:10,000 (#691001, MP Biomedicals, Illkirch, France) or VINCULIN (monoclonal mouse anti-vinculin antibody, 1:5000, V9131, Sigma–Aldrich, Taufkirchen, Germany) levels were used to ensure an equal sample loading. Membranes were washed with TBST and incubated for 1 h with the appropriate fluorescent-secondary antibody, dilution depending on primary antibody (LI-COR, Bioscience, Bad Homburg, Germany). After rewashing with TBST detection was performed using Odyssey LX Infrared Imaging System (LI-COR Bioscience, Bad Homburg, Germany). The analysis and densiometric quantification of the immunoblots was performed using the Image Studio Software (v5.2.5, LI-COR Bioscience, Bad Homburg, Germany) including the normalization to the loading control ß-ACTIN. The uncropped western blot figures can be found in the Supplementary Materials.

### 2.4. Total RNA Isolation, cDNA Synthesis and Quantitative Real-Time PCR

Total cellular RNA was isolated by using the NucleoSpin RNA II kit (Macherey-Nagel, Düren, Germany). The RNA (1 µg) was reversely transcribed by using the RevertAid RT kit (Thermo scientific, Offenbach, Germany) according to the manufacturer’s instructions. Samples were analyzed in triplicates on a Corbett Research Rotor-Gene 6000 real-time PCR instrument (Corbett Life Science, Mortlake, Australia) using the qPCR SYBR Green No-ROX Mix SensiFAST (Bioline, London, UK). For analysis, the mRNA expression levels were normalized to those of L32. Primer sequences were designed by Primer-BLAST (NCBI) and produced by ThermoFisher Scientific (Offenbach, Germany) or from Eurofins (Eurofins Genomics Germany GmbH, Ebersberg, Germany). For primer sequences see Table 2.

### 2.5. Murine HCC Samples

Murine HCC Samples were kindly provided by the Dauch group (Tuebingen, Germany) and generated as previously described [26,27].

### 2.6. RNA Isolation and Affymetrix mRNA Microarray

In this study we used an Affymetrix Microarray dataset derived from control siRNA (AS) transfected HepG2 cells treated either with DMSO or Nutlin for 24 h. Total RNA was isolated using the TRIzol method according to the manufacturer’s protocol (Invitrogen, Karlsruhe, Germany). RNA was reverse transcribed using the SuperScript Choice System (Invitrogen, Karlsruhe, Germany) according to the manufacturer’s protocol. With the ENZO BioArray HighYield RNA Transcript Labeling Kit (Enzo Life Sciences, Farmingdale, NY, USA) Biotin-labeled cRNA was produced. For the in vitro transcription (IVT) 3.3 μL of cDNA was used (standard protocol, Affymetrix, Santa Clara, CA, USA) followed by a cleanup using CHROMA SPIN-100 columns (ClonTech, Mountain View, CA, USA). cRNA was quantified by Spectrophotometric analysis (A260/A280 ratios, ranging from 1.9 to 2.1). Gene expression profiling was performed using HuGene10_st-v1-type arrays (Affymetrix). Following working steps (cDNA/cRNA fragmentation, synthesis and hybridization to arrays) were performed according to the recommendations of the manufacturer. For the annotation of the arrays a custom CDF Version 22 with Entrez based gene definitions was used. To normalize the raw fluorescence intensity values a quantile normalization was applied.

### 2.7. MTT-Assay

Cell culture and transfection with the indicated siRNAs were performed as described above. After 48 h cells were trypsinized, counted and re-seeded in a 96-well plate at a density of 5.000 (HLE, HLF) and 10.000 (HepG2) cells per well in 100 µL of medium and incubated at 37 °C in an atmosphere containing 5% CO_2_ for 48 h. In a next step 20 µL of MTT-Solution (5 mg/mL Thiazolyl Blue Tetrazolium Bromid (Sigma, Taufkirchen, Germany) in 1× PBS (pH 7,4)) were added and the plate was further incubated at 37 °C for 4 h. Next 100 µL of 10% SDS (Carl Roth GmbH und Co. KG, Karlsruhe, Germany) were added to each well to solubilizes cells. After 72 h absorption was measured at 595 nm using an iMARK^TM^ microplate reader (Bio-Rad Laboratories Inc., Munich, Germany). For each condition three technical replicates were averaged and normalized to their corresponding controls.

### 2.8. Prediction of a Non-Canonical FOXM1-Binding Site in HELLS

Publicly available ChIP-Seq data for FOXM1 at ENCODE (www.encodeproject.org accessed on 18 November 2021) was queried via the Genome Browser (https://genome.ucsc.edu accessed on 18 November 2021). ChIP-Seq peaks within *HELLS* sequence were examined for the non-canonical FOXM1 binding element named CHR (cell cycle genes homology region) as reported by Chen et al. [25]. An intronic region between exon 1 and exon 2, covered by a ChIP-Seq peak and containing two CHR motives (DTTYRAA) in close proximity was examined after FOXM1 Chromatin immunoprecipitation.

### 2.9. FOXM1 Chromatin Immunoprecipitation

Forkhead Box Protein M1 (FOXM1) Chromatin immunoprecipitation (ChIP) in HepG2 and HLF cells was performed as previously described by Weiler et al. [28].

### 2.10. Statistical Analyses and Software

The shown data are presented as the mean of at least three independent experiments including standard deviation (stdv.), unless otherwise specified. Statistical significance was evaluated by Student’s *t*-test using Excel 2019 (Microsoft, Redmond, WA, USA). Correlation data (correlation coefficient determined by Spearman Rank Test) and Box Plot Data (log2FC (fold change) ≥ ±1.0; *p*-value ≤ 0.05; Box-plot elements are center line: Median; box limits: Upper and lower quartiles) as well as Kaplan Meier Data based on the TCGA data set and generated by GEPIA2 [29].

## 3. Results

### 3.1. HELLS Is a Repression Target of P53

**Figure 1:**
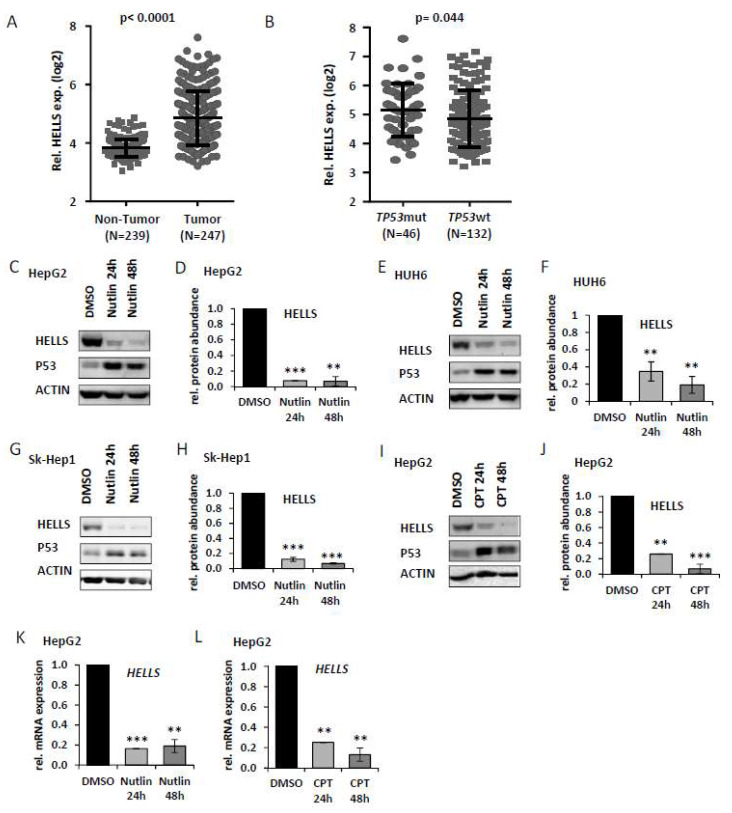
HELLS expression levels are down regulated after P53 induction: (**A**) *HELLS* expression in hepatocellular carcinoma (HCC, Tumor) and matching non-tumorous tissue (NT). (**B**) *HELLS* expression in HCC harboring either wildtype (wt) or mutated (mut) *TP53*. Analysis of the data set has been performed as previously published by Roessler et al. [30]. Statistical significance was determined using the Mann-Whitney test. *p*-values ≤ 0.05 were considered significant. (**C**) HepG2 cells were treated with DMSO or Nutlin-3a for 24 h or 48 h. Cell extracts were analyzed by immunoblotting with indicated antibodies. (**D**) Relative densitometric quantification of immunoblots derived from three independent experiments and normalized to the DMSO control as described in (**C**). (**E**,**F**) HUH6 cells treated as described in (**C**,**D**). (**G**,**H**) Sk-Hep1 cells treated as described in (**C**,**D**). (**I**) HepG2 cells were treated with DMSO or CPT for 24 h or 48 h. Cell extracts were analyzed by immunoblotting with indicated antibodies. (**J**) Relative densitometric quantification of immunoblots derived from three independent experiments and normalized to the DMSO control as described in (**I**). (**K**) HepG2 cells were treated with DMSO or Nutlin-3a for 24 h or 48 h. *HELLS* mRNA was quantified by qRT-PCR. Standard deviations are derived from three biological replicates. (**L**) HepG2 cells were treated with DMSO or CPT for 24 h or 48 h. *HELLS* mRNA was quantified by qRT-PCR. Standard deviations are derived from three biological replicates. ** *p* < 0.01, *** *p* < 0.001 (Student’s *t*-test). Data are presented as mean ± stdv.

Law et al. [19] described overexpression of *HELLS* in liver cancer and particularly high expression levels in *TP53* mutant tumors, which we could confirm in another HCC cohort [30] (Figure 1A,B). Thus, we hypothesized that *HELLS* may represent a P53 repression target. To test this hypothesis, we treated HepG2 cells (containing wildtype P53, P53wt) with Nutlin-3a for 24 h and 48 h. Nutlin-3a stabilizes P53 through disruption of the MDM2-P53 interaction protecting it from degradation and leading to its accumulation. As shown in Figure 1C,D there was a striking decrease (~90%) of HELLS protein upon Nutlin-3a treatment in HepG2 cells as evaluated by immunoblotting and corresponding densitometric quantification. A similar effect was observed in HUH6 and Sk-Hep1 cells (also harboring P53wt, respectively), under the same conditions (Figure 1E–H) and in HepG2 treated with Camptothecin (CPT), a topoisomerase inhibitor inducing DNA double strand breaks (Figure 1I–J). We then determined if the decrease of HELLS protein upon P53 activation is also detectable at the mRNA level by using qRT-PCR (for Primer sequences see Table 2). Indeed, as illustrated in Figure 1K,L we observed dramatically reduced *HELLS* transcript levels in HepG2 cells either treated by Nutlin-3a or CPT for 24 h or 48 h. These data indicate that *HELLS* is strongly repressed at the transcript and protein level upon P53 induction in a DNA damage-dependent and -independent setting.

**Figure 2:**
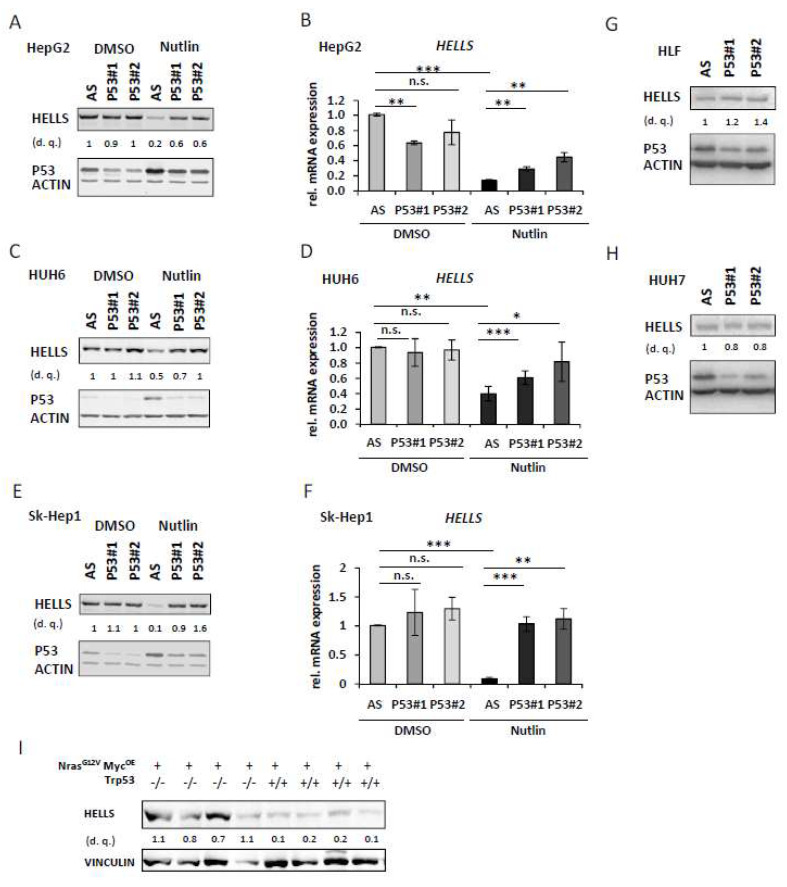
HELLS represents a repression target of P53wt in vitro and in vivo: (**A**) HepG2 cells were transfected with either control siRNA (AS) or two different *TP53* siRNAs (P53#1 and #2) and treated with DMSO or Nutlin-3a for 24 h. Cell extracts were analyzed by immunoblotting with indicated antibodies. Densitometric quantification (d. q.) of HELLS normalized to control siRNA AS DMSO. (**B**) Relative *HELLS* transcript levels derived from four biological replicates of the same conditions as described in (**A**) were measured by qRT-PCR. (**C**) HUH6 cells as described in (**A**). (**D**) Relative *HELLS* transcript levels derived from three biological replicates of the same conditions as described in (**C**) were measured by qRT-PCR. (**E**) Sk-Hep1 cells treated as described in (**A**). (**F**) Relative *HELLS* transcript levels derived from three biological replicates of the same conditions as described in (**E**) were measured by qRT-PCR. (**G**) HLF cells were transfected with either control siRNA (AS) or two different *TP53* siRNAs (P53#1 and #2) and harvested 72 h after transfection. Cell extracts were analyzed by immunoblotting with indicated antibodies. Densitometric quantification of HELLS normalized to control siRNA. (**H**) HUH7 cells as described in (**G**). (**I**) Nras- and Myc-driven murine HCC either developed in a *Trp53−/−* or *Trp53*+/+ background. Murine HCC tissues were analyzed by immunoblotting with the indicated antibodies. Densitometric quantification of HELLS for each sample. * *p* < 0.05, ** *p* < 0.01, *** *p* < 0.001, n.s. = not significant (Student’s *t*-test). Data are presented as mean ± stdv.

To validate that the observed effects under Nutlin-3a treatment are indeed P53-dependent (and not unspecific effects of the compound) we transfected HepG2, HUH6 and Sk-Hep1 cells either with a control siRNA (Allstars, AS) or with two different *TP53*-specific siRNAs and added either DMSO or Nutlin-3a. As displayed in Figure 2A–F we observed (depending on the cell line) a complete or nearly complete rescue of HELLS repression at the protein and mRNA-level in the *TP53* siRNA transfected conditions upon Nutlin-3a treatment, consistent with a P53-dependent effect. Transfection with the control siRNA AS had no significant effects on either P53 or HELLS protein level compared to the untransfected conditions (Figure S1A–I).

Since repression targets of P53wt can also represent activation targets of ‘gain of function’ P53 mutants, we determined if knockdown of mutant P53 (P53mut) in HLF (P53^G244A^) mutants and HUH7 (P53^Y220C^) affects HELLS protein expression. However, as shown in Figure 2G, H HELLS protein remained unaltered by knockdown of P53mut further suggesting the negative regulation of HELLS by P53wt and not by the aforementioned P53 mutants (with the caveat that the knockdown efficiency of P53mut was lower than for P53wt). To further substantiate the impact of P53wt on HELLS expression in vivo we evaluated tumor samples derived from N-RAS and MYC-driven murine HCCs either developed in a *Trp53−/−* or a *Trp53*+/+ background by immunoblotting. As demonstrated in Figure 2I HELLS protein expression was higher in *Trp53−/−* tumors compared to *Trp53*+/+ tumors. These data suggest that *HELLS* represents a repression target of P53wt in vitro and in vivo.

**Figure 3:**
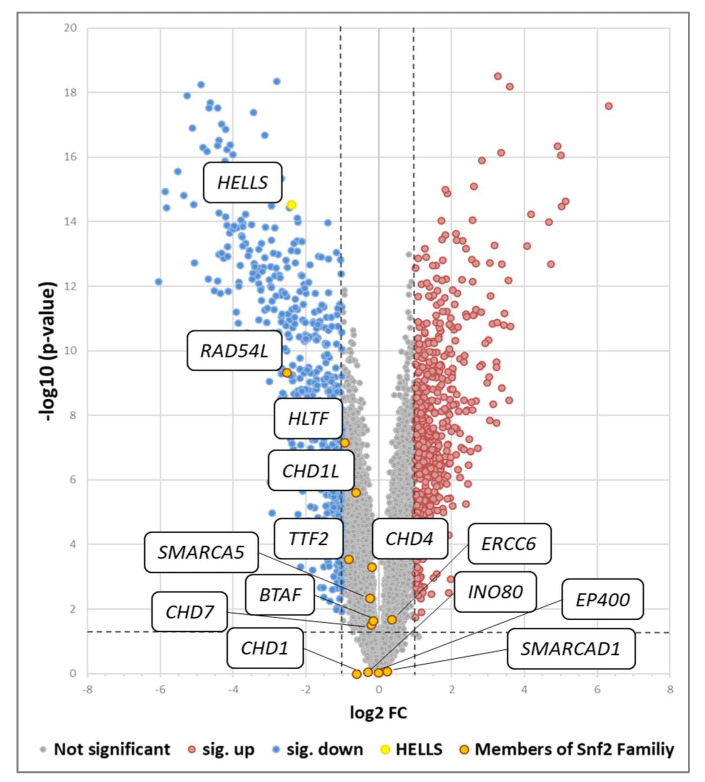
P53-mediated *HELLS* repression is largely selective within the SNF2-like helicase family. Volcano plot shows 9129 transcripts identified in HepG2 cells by Affymetrix microarrays. After 24 h Nutlin-3a treatment, 530 transcripts were significantly upregulated (colored in red) and 571 transcripts were significantly down regulated (colored in blue). (Horizontal dotted line *p* = 0.05; vertical dotted lines log_2_ fold-change 1.0 or −1.0). mRNA levels of SNF2-like family members are highlighted in orange including *HELLS* in yellow.

To determine to what extent repression of *HELLS* by P53 is selective within the HELLS containing SNF2 family of helicase-related genes we performed gene expression arrays in HepG2 cells treated either with DMSO or Nutlin-3a for 24 h. Consistent with the aforementioned data, *HELLS* mRNA was strikingly reduced upon Nutlin-3a treatment (log2 fold change of −2.39, *p*-value of 2.90 × 10^−15^) and besides *RAD54L* no other member of the SNF2 family of helicase-related genes (*HLTF*, *CHD1L*, *TTF2*, *CHD4*, *SMARCA5*, *ERCC6*, *BTAF*, *INO80*, *CHD7*, *CHD1*, *EP400*, *SMARCAD1*) was significantly downregulated based on the threshold of log2 fold change ≥/≤ ±1.0 and significance value of *p*-value ≤ 0.05 (Figure 3). We also performed qRT-PCR analyses of HepG2 cells either transfected with control (AS) or *TP53* siRNA (P53#1 and #2) and treated with either DMSO or Nutlin-3a to quantify transcript changes of SNF2-family members. Consistent with the transcriptome data only *RAD54L* showed significantly decreased mRNA levels upon Nutlin-3a treatment, which could be completely rescued in both corresponding *TP53* siRNA treated conditions, in contrast to *HLTF*, *CHD1L* and *SMARCA5* (Figure S2A–D). Together these data demonstrate that P53-mediated repression is most prominent for *HELLS* and with the exception of *RAD45L* selective within the SNF2 helicase family.

Figure S3 highlights well known P53 activation (e.g., *MDM2*, *CDKN1A* and *TIGAR*) and repression (e.g., *CDC25C*, *CCNB1* and *PLK1*) targets [31] as a plausibility check of the data set.

### 3.2. Repression of HELLS by P53 Involves P21 Induction

**Figure 4:**
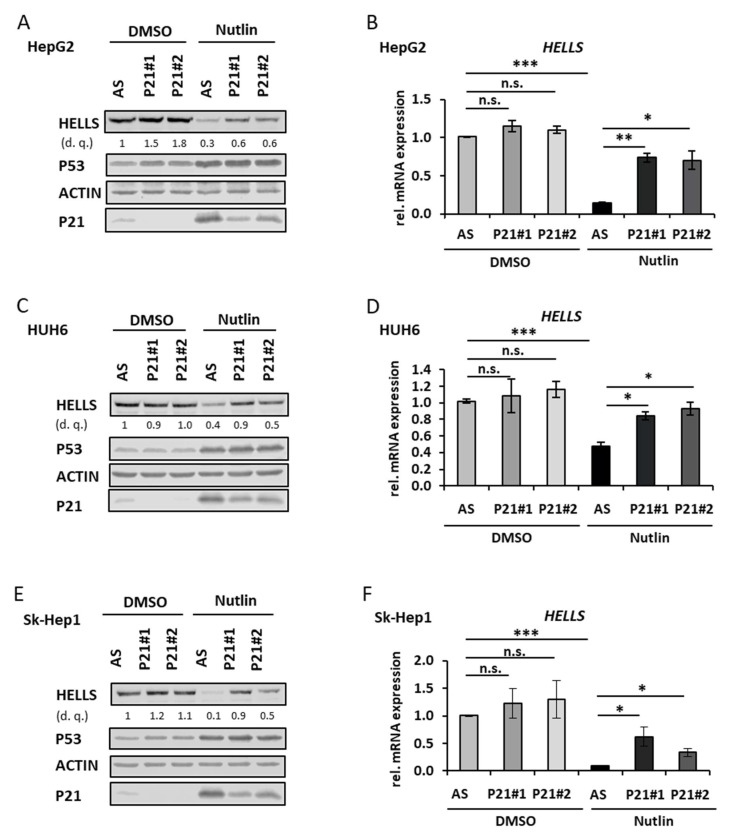
P53 induced HELLS repression is P21 dependent. (**A**) HepG2 cells were transfected with either control siRNA (AS) or two different P21/*CDKN1A* siRNAs (P21#1 and #2) and treated with DMSO or Nutlin-3a for 24 h. Cell extracts were analyzed by immunoblotting with indicated antibodies. Densitometric quantification (d. q.) of HELLS normalized to control siRNA DMSO. (**B**) Relative *HELLS* transcript levels derived from three biological replicates of the same conditions as described in (**A**) were measured by qRT-PCR. (**C**) HUH6 cells as described in (**A**). (**D**) Relative *HELLS* transcript levels derived from three biological replicates of the same conditions as described in (**C**) were measured by qRT-PCR. (**E**) Sk-Hep1 cells as describes in (**A**). (**F**) Relative *HELLS* transcript levels derived from three biological replicates of the same conditions as described in (**E**) were measured by qRT-PCR. * *p* < 0.05, ** *p* < 0.01, *** *p* < 0.001, n.s. = not significant (Student’s *t*-test). Data are presented as mean ± stdv.

Transcriptional repression of target genes by P53 frequently involves P21 [12]. We therefore tested if P21 depletion is sufficient to reverse *HELLS* repression upon P53 induction in HepG2, HUH6 and Sk-Hep1 cells. Figure 4A–F illustrates that downregulation of HELLS protein and mRNA by P53 was (depending on the cell line) either partially or completely rescued in the P21 knockdown conditions under Nutlin-3a treatment indicating that P53 induced repression of *HELLS* is at least partially mediated by P21.

### 3.3. P53/P21 Dependent HELLS Repression Requires Downregulation of FOXM1

**Figure 5:**
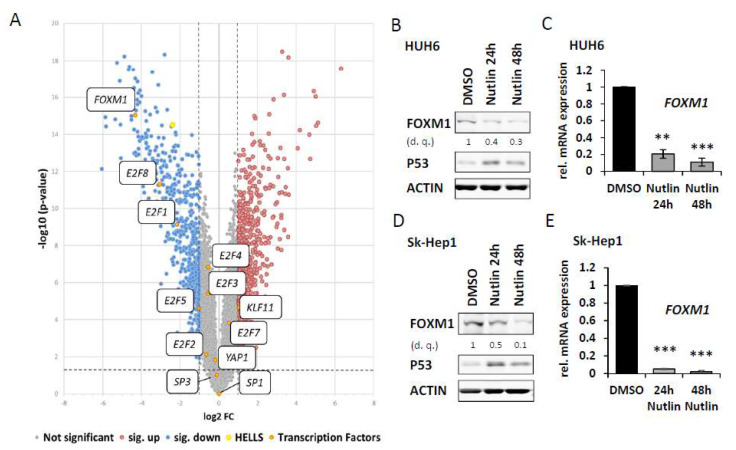
*FOXM1* is repressed after P53 induction. Research for transcription factors involved in P53/P21-dependent repression of *HELLS* reveals *FOXM1*: (**A**) Volcano plot as introduced in Figure 3. Transcription factors previously described as regulators/interactors of *HELLS* are highlighted in orange. FOXM1 as the most strikingly repressed transcription factor with potential involvement in *HELLS* regulation and high functional relevance in HCC (**B**) HUH6 cells were treated with DMSO or Nutlin-3a for 24 h or 48 h. Densitometric quantification (d. q.) of FOXM1 normalized to DMSO. (**C**) Relative *FOXM1* transcript levels derived from three biological replicates of the same conditions as described in (**B**) were measured by qRT-PCR. (**D**) Sk-Hep1 cells as described in (**B**). (**E**) Relative *FOXM1* transcript levels derived from three biological replicates of the same conditions as described in (**D**) were measured by qRT-PCR. ** *p* < 0.01, *** *p* < 0.001 (Student’s *t*-test). Data are presented as mean ± stdv.

Next, we set out to identify transcription factor(s) (TF) possibly involved in P53/P21-dependent repression of HELLS. To do so, we queried the aforementioned transcriptomic data set (introduced in Figure 3) for downregulated transcription factors with potential relevance in this context. Interestingly, SP1 [19], E2F3 [32] and YAP1 [33] previously reported as important regulators/interactors of HELLS and E2F7 as an important mediator of P53 target gene repression [34,35] were not altered at the transcript level under Nutlin-3a treatment. However, as the most strikingly downregulated TF with a link to P53/P21 [36], and high relevance in liver cancer [37,38,39,40] we identified *FOXM1* (Figure 5A and Table S1). Consistent with previous findings in HepG2 [36] we could validate a strong downregulation of FOXM1 protein and mRNA upon Nutlin-3a treatment also in HUH6 and Sk-Hep1 cells (Figure 5B–E).

**Figure 6:**
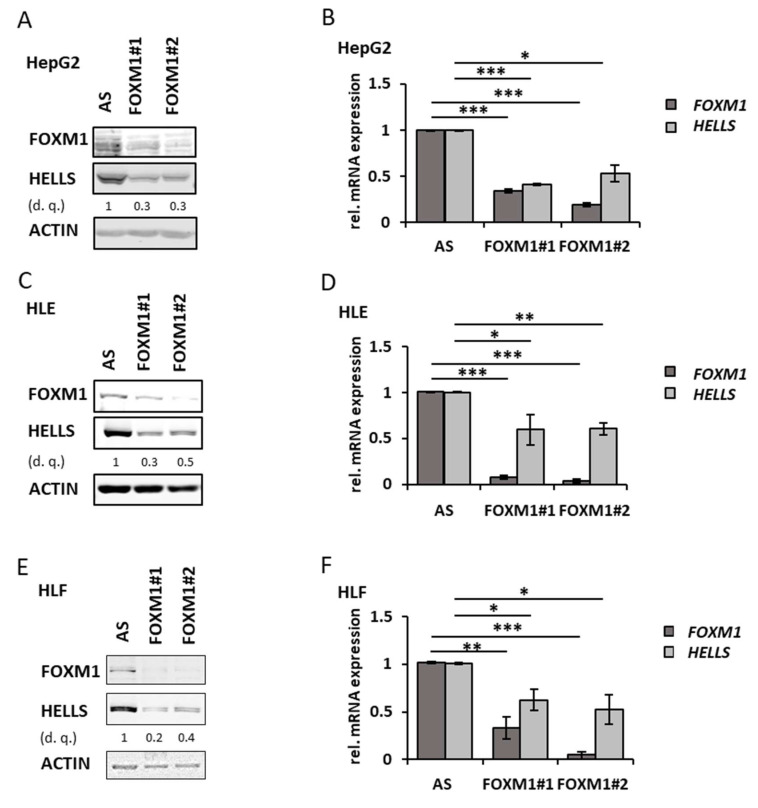

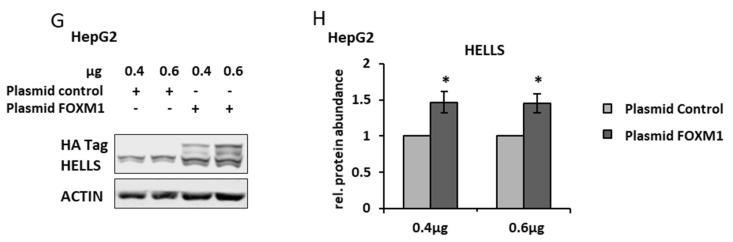
P53/21 dependent HELLS repression requires downregulation of FOXM1: (**A**) HepG2 cells were transfected with either control siRNA (AS) or two different FOXM1 siRNAs (FOXM1#1 and #2). Cell extracts were analysed by immunoblotting with indicated antibodies. Densitometric quantification (d. q.) of HELLS normalized to control siRNA. (**B**) HepG2 cells as described in (**A**) with *HELLS* and *FOXM1* transcript levels measured by qRT-PCR. Standard deviations are derived from three biological replicates. (**C**) HLE cells as described in (**A**). (**D**) HLE cells as described in (**C**) with *HELLS* and *FOXM1* transcript levels measured by qRT-PCR. Standard deviations are derived from three biological replicates. (**E**) HLF cells as described in (**A**). (**F**) HLF cells as described in (**E**) with *HELLS* and *FOXM1* transcript levels measured by qRT-PCR. Standard deviations are derived from three biological replicates. (**G**) HepG2 cells were transfected with either a control or FOXM1 plasmid in different concentrations as indicated and were subjected to immunoblotting with indicated antibodies. (**H**) Relative densitometric quantification of immunoblots derived from three independent experiments and normalized to the DMSO control as described in (**G**). * *p* < 0.05, ** *p* < 0.01, *** *p* < 0.001 (Student’s *t*-test). Data are presented as mean ± stdv.

To further support that FOXM1 regulates *HELLS* expression we performed transfection experiments using two *FOXM1*-specific siRNAs, which resulted in a decrease of HELLS protein and transcript in HepG2, HLE and HLF cells (Figure 6A–F). Moreover, as demonstrated in Figure 6G,H exogenously expressed HA-tagged FOXM1 from an expression construct (pDEST) resulted in an increase of HELLS protein compared to the empty vector transfected control condition in HepG2 cells. To substantiate that FOXM1 directly regulates *HELLS* expression we performed FOXM1 chromatin immunoprecipitations (ChIP) in HepG2 and HLF cells. By in silico analyses using publicly available ChIP-Seq data for FOXM1 at ENCODE we identified a potential non-canonical FOXM1 binding site containing two CHR motifs in the first intron in close proximity as described by Chen et al. [25]. For the subsequent qRT-PCR we used a primer pair (*HELLS*_CHR) to amplify the region containing the two CHR motifs and another primer pair (control) to amplify a region within the 3′UTR of the *HELLS* gene serving as negative control (Figure S4A). As shown in Figure S4B,C, we obtained signals up to three times higher by using the *HELLS*_CHR primer pairs compared to the negative control. We conclude from this data that FOXM1 directly regulates *HELLS* expression by binding to an intronic non-canonical binding site in the 1st intron of the *HELLS* gene. In addition, knockdown of *FOXM1* in HepG2, HLE and HLF cells resulted in decreased cell viability (Figure S5A–C) consistent with previously published data for FOXM1 [23] and recapitulating a functional effect of *HELLS* depletion as demonstrated by Law et al. [19]. We conclude that P53/P21-dependent *HELLS* repression involves downregulation of FOXM1, itself being a functionally relevant direct positive transcriptional regulator of *HELLS* in HCC.

### 3.4. HELLS and FOXM1 mRNA Correlates in Human HCC Cohorts

**Figure 7:**
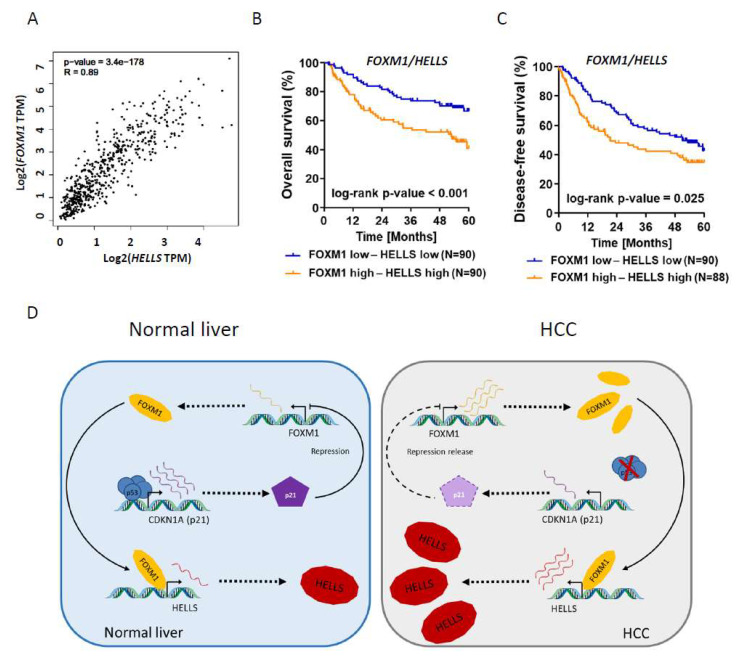
Statistical analysis of *HELLS* and *FOXM1* in various aspects. (**A**) Correlation between *HELLS* and *FOXM1* expression in human HCC based on a TCGA data set. (**B**) Overall survival of HCC patients with high-high or low-low expression of *HELLS* and *FOXM1* based on a previously published transcriptomic data set by Roessler et al. [30]. (**C**) Disease-free survival of HCC patients with high-high or low-low expression of *HELLS* and *FOXM1* based on a previously published transcriptomic data set by Roessler et al. [30] (**D**) Illustration of the regulatory link between P53 and HELLS: Normal liver tissue (left panel): P53 induction leads to reduced *HELLS* expression via P21-dependent repression of *FOXM1*. In HCC (right panel): Non-functional P53 (either mutated or functionally inactivated) results in a repression relieve leading to *FOXM1* overexpression and consecutively *HELLS* overexpression.

To further substantiate our in vitro findings, we tested if *HELLS* and *FOXM1* expression are correlated to each other in human HCC tissue samples. By including two different HCC cohorts (TCGA (LIHC) and Roessler et al. [30]) we found in each cohort a strong and highly significant correlation between *HELLS* and *FOXM1* transcript levels (r = 0.89, *p* ≤ 0.001 and r = 0.64 *p* ≤ 0.001), as shown in Figure 7A and Figure S6A. Consistent with previously published data both *HELLS* and *FOXM1* were significantly overexpressed in HCC samples compared to non-tumorous liver tissue (Figure 1A (*HELLS*) and Figure S6B (*FOXM1*)) as well as in *TP53* mutant compared to *TP53* wt HCCs (Figure 1B (*HELLS*) and Figure S6C (*FOXM1*), while P21/*CDKN1A* was lower expressed in *TP53* mutant HCCs compared to *TP53* wt HCCs (Figure S6D). In line with both factors reported to be independent negative prognostic markers [19,24] combined higher than median expression of *HELLS* and *FOXM1* was clearly associated with poorer overall and disease-free survival in HCC patients (Figure 7B,C). Finally, by comparing tumor (T) and non-tumorous tissue (NT) tissue in the TCGA HCC (LIHC) cohort we only found overexpression of *HELLS*, but not other members of the SNF2 family in HCC based on log2 fold change cut off of ≥/≤ ±1.0 and *p*-value cut off of ≤0.05 (Figure S7).

As summarized in Figure 7D our data suggest that *HELLS* is regulated by FOXM1 itself being suppressed by P53 in a P21-dependent manner under physiological conditions (left panel). Accordingly, functional or mutational inactivation of P53 followed by loss of P21 induction results in overexpression of *FOXM1* that in turn drives overexpression of *HELLS* in hepatocarcinogenesis. Moreover, key findings such as P53-dependent repression of HELLS and the strong correlation of *HELLS* and *FOXM1* could be recapitulated in lung and breast cancer cell lines/patient data (Figure S8). These data suggest that the aforementioned regulatory mechanism is transferable to other tumor entities and is not restricted to HCC.

## 4. Discussion

In this study, we could show that *HELLS* is a strong repression target of P53 in liver cancer in vivo and in vitro. This finding perfectly fits to the pro-tumorigenic effects of HELLS in promoting tumor cell growth, migration and in vivo tumorigenicity, described by Law et al., who also reported a correlation of *HELLS* with early onset age, direct liver invasion, venous invasion, advanced TNM stages, higher histological grade, Ki67-proliferation index, as well as with poorer overall survival and disease-free survival [19]. Thus, negative regulation of *HELLS* reflects an additional important aspect of P53′s ability to suppress tumor development and progression in liver cancer (and presumably also in other tumor entities). While this study provides evidence for the important role of P21 and FOXM1 as relevant mediators of P53-dependent repression of *HELLS*, other genes also being regulated directly or indirectly by P53 may also participate in the regulation of *HELLS* in this context.

Typical P53 tumor suppressive responses are cell cycle arrest and apoptosis [5] in both of which *HELLS* repression could be important. Nutlin-3a treatment results in a G2/M and G1 arrest in HepG2 cells [36] and at the same time is associated with strong repression of *HELLS* suggesting that negative regulation of *HELLS* might be linked to P53-mediated cell cycle regulation. In addition, direct *HELLS* depletion is associated with increased apoptosis in liver cancer cells [19]. As we observed strong repression of *HELLS* upon CPT treatment, well documented to result in a pro-apoptotic P53 response, these findings together suggest that reduced *HELLS* expression also contributes to P53 induced apoptosis. A further link to apoptosis is given by the fact that among previously reported HELLS repressed tumor suppressor genes (e.g., *RASAL1*, *IGFBP3*, *CDH1*, *CREB3L3*) [19]. we found *RASAL1* being significantly upregulated in our transcriptomic data set of Nutlin-3 treated HepG2 cells (Figure S9). The Ras GTPase activating protein RASAL1 has been studied in the context of stellate cells and liver fibrosis [41] and has been characterized as an important tumor suppressor by negatively regulating RAS protein signal transduction in HCC [42]. As such, in the absence of RAS mutations reactivation of RASAL1 inhibits proliferation and induces apoptosis in HCC [42]. These functions of RASAL1 are therefore well compatible with P53-dependent repression of the repressor HELLS resulting in a net induction of RASAL1 within a pro-apoptotic P53 response. However, the fact *IGFBP3*, *CDH1* and *CREB3L3* (Figure S9) remain unaltered in this setting indicates that HELLS repression targets do not necessarily represent P53 activation targets.

Besides cell cycle regulation and apoptosis, metabolic alterations can also represent P53 response(s). *HELLS* knockdown has been linked to metabolic changes indicating reversion of the Warburg effect [19,43]. Thus, repression of HELLS by P53 could also play a role in P53′s well documented ability to block the Warburg effect by inhibiting glycolysis and promoting oxidative phosphorylation via NF-κB [44,45]. Another example of negative gene regulation by P53 in a P21-dependent manner affecting metabolism in liver cancer cells is the repression of guanosine monophosphate synthetase (GMPS) as a key enzyme of the purine biosynthesis pathway. Repression of *GMPS* resulted in reduced cell viability and induced cellular senescence [46]. Moreover, direct knockdown of *GMPS* was followed by P21 upregulation suggestive of a positive feedback loop between P53/P21 and GMPS.

A potential feedback loop between P53/P21 and HELLS in a metabolic context could also be envisioned based on the findings of Chen et al. [47]. The authors demonstrated in nasopharynx carcinoma and lung carcinoma cell lines that HELLS stabilizes P53 post-transcriptionally by preventing it from proteasomal degradation and promoting its phosphorylation to orchestrate cancer cell lipid metabolism. However, as shown in Figure S10, we did not observe a striking and consistent decrease of basal P53 levels upon knock down of *HELLS* using two different siRNAs in three different cancer cell lines containing P53wt (HepG2, HUH6 and Sk-Hep1). One explanation for these results could be cell type-specific effects (liver derived cell lines used by us vs. nasopharynx and lung carcinoma cell lines used by Chen et al.). Moreover, technical and time-dependent aspects may also play a role in this context, since we analyzed P53 protein levels 72 h upon transient siRNA transfection, while Chen et al. evaluated P53 protein of a stable shRNA-mediated *HELLS* knockdown.

P53 controls a large gene expression network that determines cell fate by up- and downregulation of specific mRNAs [12] with P53 activation targets being traditionally studied much more extensively. In fact, to what extent P53 can serve as a direct transcriptional repressor is a longstanding matter of debate [12]. A variety of different models of direct target gene repression by P53 have been proposed in the past such as recruitment of co-repressors and imperfect P53-REs leading to repression activity [12,48,49]. However, the prevailing concept is that negative gene regulation by P53 occurs in an indirect fashion. In particular P21/*CDKN1A* is frequently involved in P53-dependent repression via the E2F4-Rb repressive complex or the DREAM complex or miR34a [12,50,51]. Alternatively, direct activation of E2F7 by P53 as a repressive E2F transcription factor has also been reported [34,35]. Among the multitude of miRNAs induced by P53, miR34a is probably the best characterized miR regarding P53 associated repression particularly of cell cycle regulating genes via the E2F pathway [12,51,52]. Even a liver tissue-specific P53-dependent repression mechanism targeting Alpha Fetoprotein (AFP, a standard tumor marker in HCC) has been described by recruiting co-(repression)factors [53]. In this context P53 activates SMAD transcription factors, which in turn bind to the AFP promoter. After associating with SnoN and the corepressor mSin3A, HDACs are recruited for chromatin remodeling resulting AFP repression [53]. In line with the dominating model of indirect negative gene regulation by P53, we identified P21 as mediator of P53-dependent repression of *HELLS* in this study and in another liver cancer-relevant context regarding the nuclear transport factors CAS/*XPO2*, *KPNA2* and *NUP155* in former studies [27,54,55].

Given the multiple pro-tumorigenic functions of HELLS (e.g., proliferation, migration, cell survival, tumorigenicity in vivo and metabolic reprogramming), strong repression of *HELLS* by P53 appears as an effective strategy to restrain tumor initiation and progression at several levels simultaneously. We believe that the importance of this repression in liver cancer is probably best supported by overexpression of *HELLS* comparing tumor and non-tumor, even more than comparing *TP53* mutant and wild-type tumors. It is estimated that a large majority of tumors contain a disrupted or at least compromised P53 tumor-suppressive network [12] not only by mutation, but also by functional inactivation through MDMX and MDM4 overexpression or viral factors (e.g., HBX [46,56]) among other mechanisms. Thus, we assume that overexpression of *HELLS* by a repression relieve upon P53 inactivation is a highly relevant mechanism in a large fraction of HCCs. This is further highlighted by the fact that *HELLS* represents the only member of the SNF2 helicase family that we found significantly overexpressed in HCC patients and was the only member (except for *RAD45L*) that we could identify as a strong P53 repression target *in vitro*. Future studies will show if HELLS could serve as a promising candidate for direct targeted therapy in HCC or as an indirect target of approaches that restore P53′s wild type function.

## Figures and Tables

**Table 1:** Sequences of siRNAs used in the study.

siRNA	Sequence
*TP53*/P53#1	UGUUCCGAGAGCUGAAUGA
*TP53*/P53#2	AAGGAAAUUUGCGUGUGGAGU
*CDKN1A*/P21#1	CAGUUUGUGUGUCUUAAUUAU
*CDKN1A*/P21#2	CUGGCAUUAGAAUUAUUUAAA
*FOXM1#1*	AUAUUCACAGCAUCAUCAC
*FOXM1#2*	GGACCACUUUCCCUACUUU
*HELLS#1*	ATGCGATGGTACCAAGTAGAA
*HELLS#2*	AAACGGTTAGGCAGAATACTA

**Table 2:** Sequences of primers used in the study.

Primer	Sequence
*CDKN1A_F*	GGCGGCAGACCAGCATGACAGATT
*CDKN1A_R*	GCAGGGGGCGGCCAGGGTAT
*L32_F*	TTCCTGGTCCACAAC
*L32_R*	TGTGAGCGATCTCG
*HELLS_F*	CAGCCATTGTGAACCGTACAA
*HELLS_R*	TCTAGTTCGTCGTTTTGGTCG
*FOXM1_F*	TGCCCAGATGTGCGCTATTA
*FOXM1_R*	TCAATGCCAGTCTCCCTGGTA
*RAD54L_F*	TCCTATGAGACCTTCCGCCT
*RAD54L_R*	AGTTCTTGAGCCTGTGTCCC
*HLTF_F*	GCTCCTCTTGTCATCCCACTCA
*HLTF_R*	CGTCTTTGCTTAGTCCATCTGCCTT
*CHD1L_F*	GGCATTCCAGACCCTCCAAA
*CHD1L_R*	GCTCCAAAAAGTGTCGCTCC
*SMARCA5_F*	AACTTACTATCCGTTGGCGATT
*SMARCA5_R*	GGTTGCTTTGGAGCTTTCTG
*CHD1_F*	CCTGGGACTCCACCTACAGA
*CHD1_R*	TGGATTCCAGAAACGGAGGC
*CREB3L3_F*	GGGCCAGTGATCCAAGTACC
*CREB3L3_R*	AGATTGCATCGTGGGGACAG
*IGFBP3_F*	TTCAGAGACTCGAGCACAGC
*IGFBP3_R*	ACAGCCGCCTAAGTCACAAA
*XAF1_F*	AGCAGGTTGGGTGTACGATG
*XAF1_R*	CCTGGCACTCATTGGCCTTA
*HELLS_3′UTR_F*	TCTTGGATACAGGCTGATGTGT
*HELLS_3′UTR_R*	ACCTAAAGCCCATGAACTGC
*HELLS_CHR_F*	CTCCAGTGCATCTCGGGTG
*HELLS_CHR_R*	GTTCAACCATTGCTGGAGCCT

## Data Availability

The data presented in this study are available in this article (and Supplementary Material). Data generated for the current study are available from the corresponding author upon reasonable request.

## Supplementary Materials

The following supporting information can be downloaded at: https://www.mdpi.com/article/10.3390/cancers14020459/s1, Figure S1: P53 and HELLS expression are not effected by control siRNA, Figure S2: P53 dependent regulation of selected Snf2 family members, Figure S3: Expression of bona fide P53 target genes upon Nutlin-3a treatment in HepG2, Figure S4: FOXM1 binds directly to HELLS, Figure S5: FOXM1 knockdown reduces viability, Figure S6: HELLS, FOXM1 and CDKN1A expression levels in human HCC, Figure S7: Expression of SNF2 family members in HCC, Figure S8: HELLS and FOXM1 expression in human breast cancer and human lung adenocarcinoma, Figure S9: Expression of previously described HELLS-repressed tumor suppressor genes upon Nutlin-3a treatment in HepG2, Figure S10: Basal levels of P53 protein are not strikingly affected by HELLS depletion, Table S1: The 50 strongest up- and downregulated genes after Nutlin-treatment of HepG2 cells.
