# Supplementary material for: HELLS Is Negatively Regulated by Wild-Type P53 in Liver Cancer by a Mechanism Involving P21 and FOXM1
Source: Cancers (Basel). 2022 Jan 17;14(2):459. doi: 10.3390/cancers14020459 (PMC8773711; doi:10.3390/cancers14020459)
Supplement: Supplementary file 1 [file cancers-14-00459-s001.zip › cancers-1564318-supplementary-1.pdf]

cancers

Supplementary material:

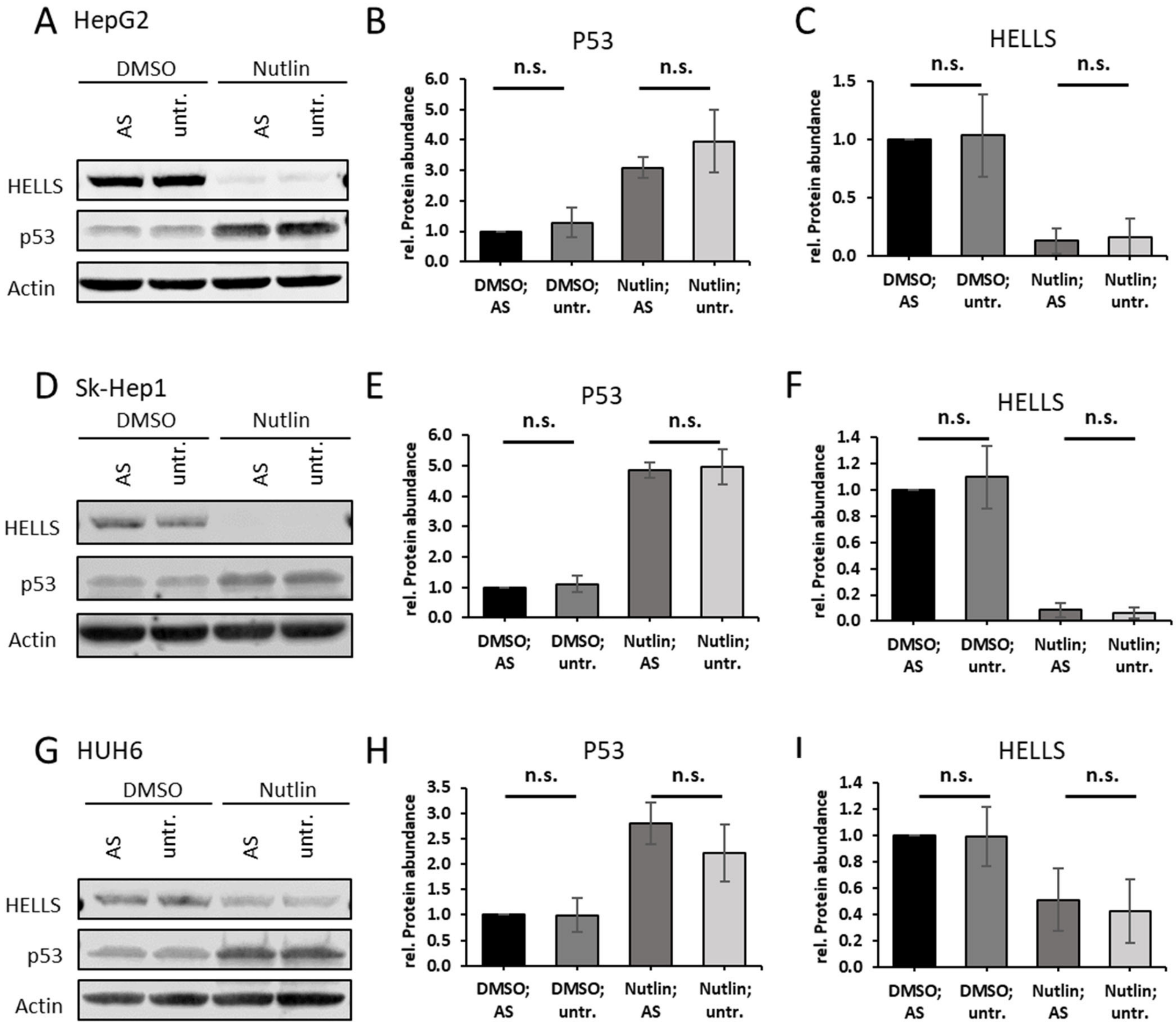

Figure S1. P53 and HELLS expression are not affected by control siRNA**.** (A) HepG2 cells were either transfected with control siRNA (AS, Allstars) or untransfected and incubated with DMSO or Nutlin-3a for 24h. Cell extracts were analyzed by immunoblotting with indicated antibodies. (B-C) Relative densitometric quantification of immunoblots derived from four independent experiments and normalized to the DMSO AS control as described in (A). (D-F) Sk-Hep1 cells were treated as described in (A) and (B). (G-I) HUH6 cells were treated as described in (A) and (B). n.s.=not significant (Student's t-test). Data are presented as mean ± stdv.

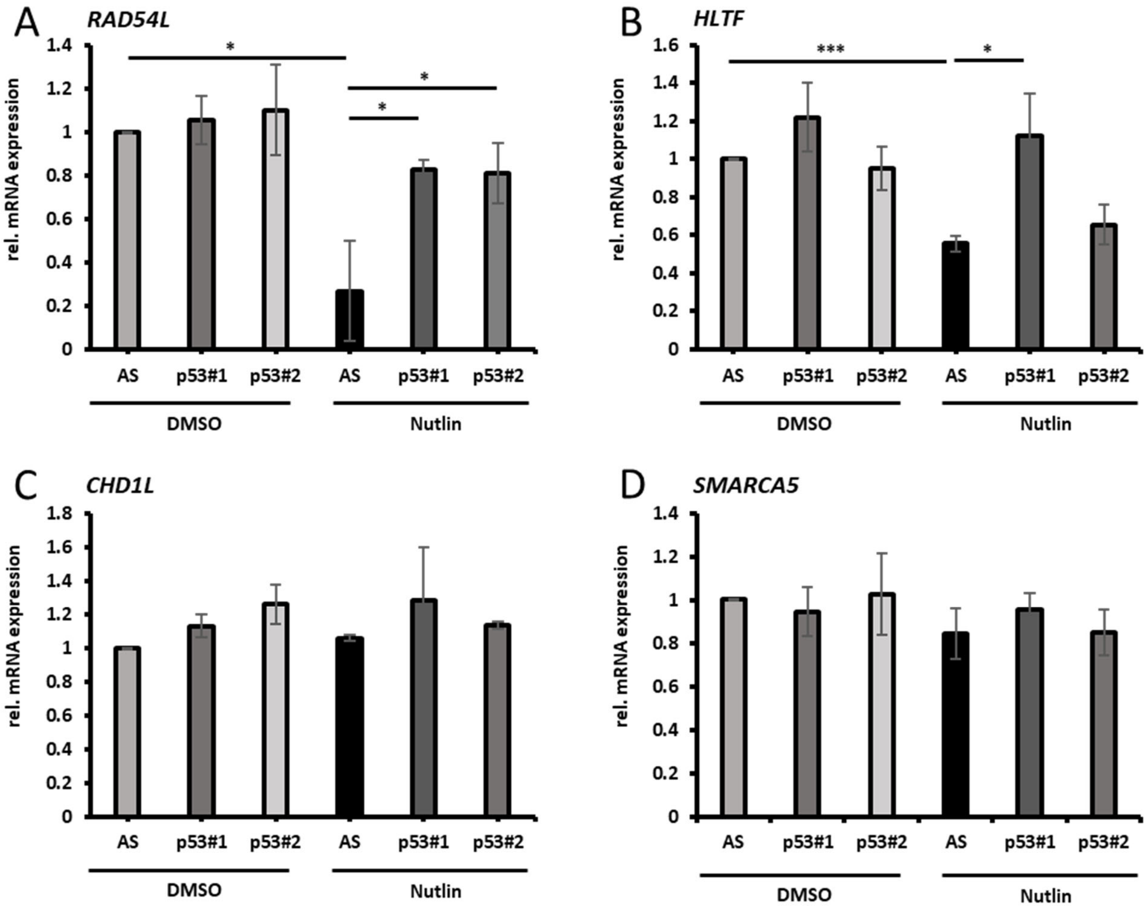

Figure S2. P53 dependent regulation of selected Snf2 family members. HepG2 cells were transfected with either control siRNA (AS) or two different P53 siRNAs (P53#1 and #2) and treated with DMSO or Nutlin-3a for 24h. Relative transcript levels of *RAD54L* (A), *HLTF* (B), *CHD1L* (C) and *SMARCA5* (D) derived from three biological replicates were measured by qRT-PCR. Significant comparisons are marked. *p < 0.05, ***p < 0.001 (Student's t-test). Data are presented as mean ± stdv.

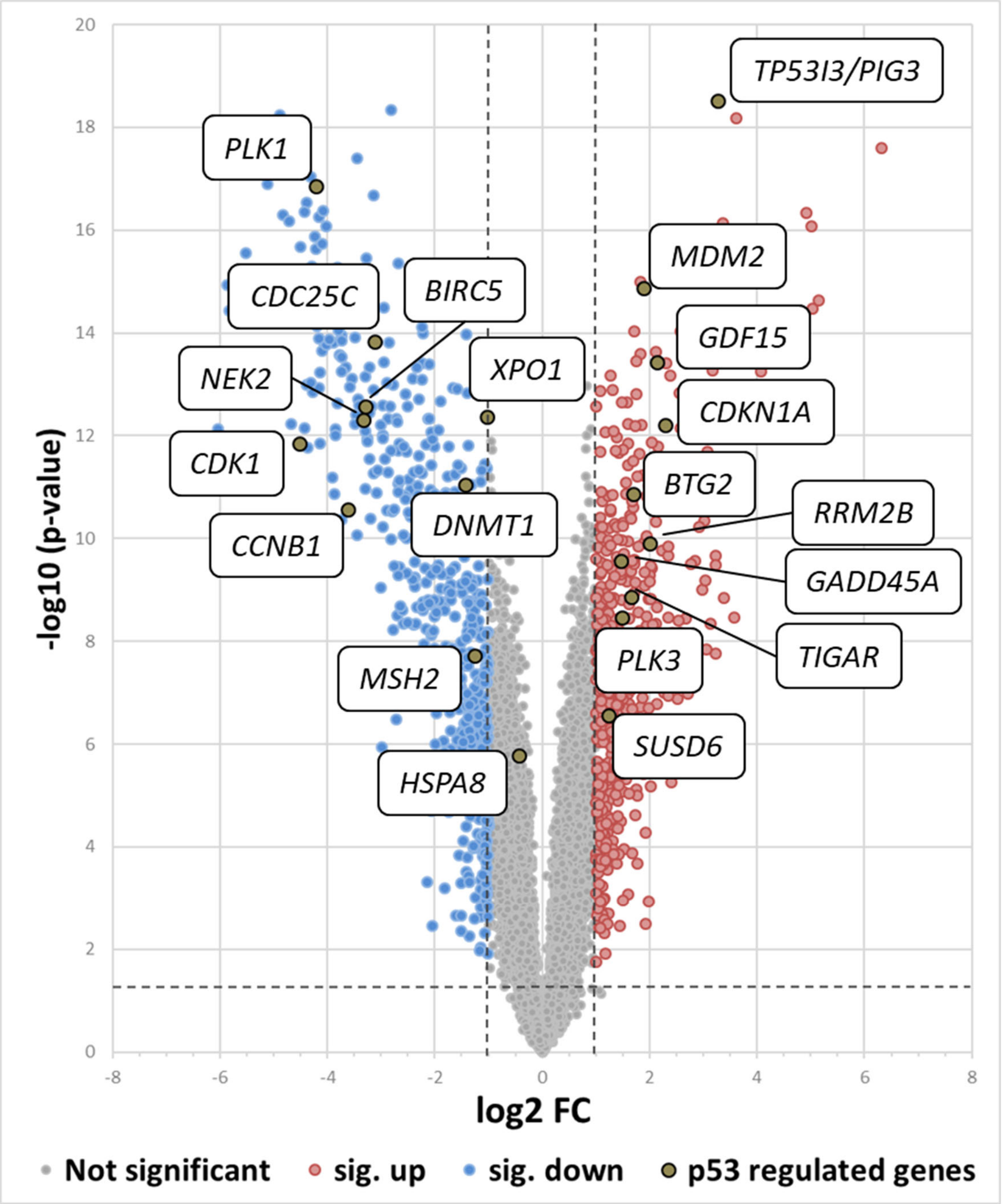

Figure S3. Expression of bona fide P53 target genes upon Nutlin-3a treatment in HepG2. Volcano plot showing 9129 transcripts identified in HepG2 cells by Affymetrix microarray. 530 transcripts were significantly up- (colored in red) and 571 transcripts were significantly down-regulated (colored in blue) after 24h Nutlin-3a treatment. (Horizontal dotted line $p = 0.05$; vertical dotted lines log2 fold-change 1.0 or −1.0). mRNA levels of previously reported P53 target genes are highlighted in olive colour.

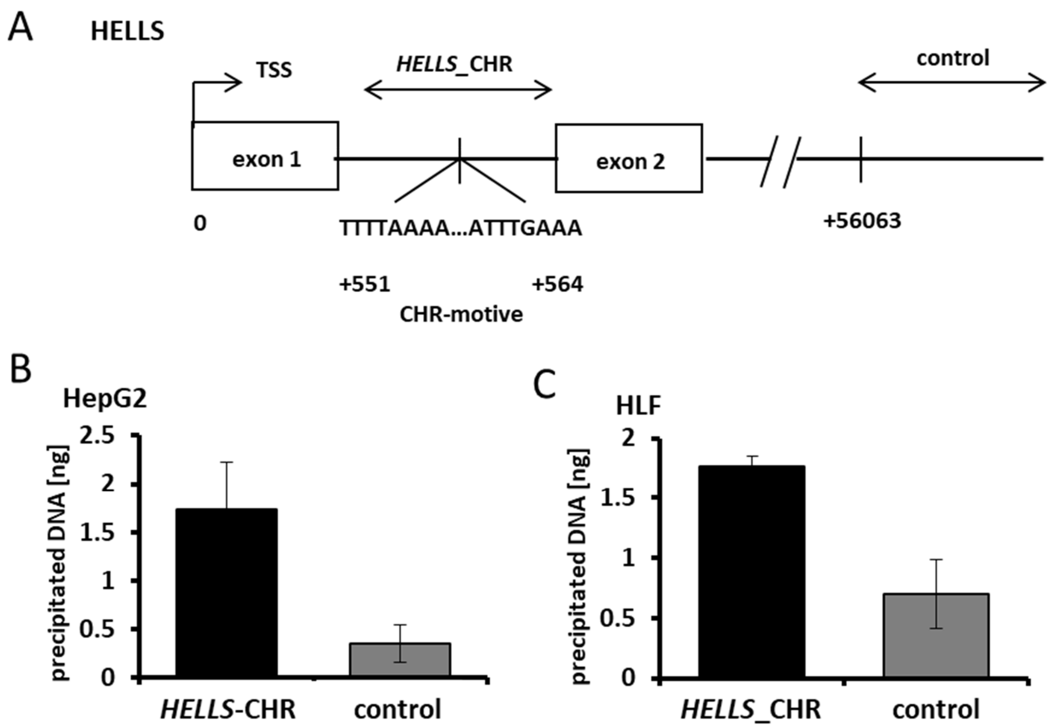

Figure S4. FOXM1 binds directly to a region in the first intron of the *HELLS* gene. (A) Schematic depiction of a non-canonical FOXM1 binding site between exon 1 and 2 containing two CHR motives as described by Chen et al. [31] Primer pair *HELLS*_CHR was used to determine FOXM1 binding, a primer pair against 3′UTR serves as control. (B) Bar diagram shows amount of DNA precipitated by FOXM1 IP in HepG2 (B) and HLF (C) cells. FOXM1 chromatin immunoprecipitation (ChIP)-qPCR and data analysis have been performed as previously described by Weiler et al. [28]. All experiments have been performed in duplicates.

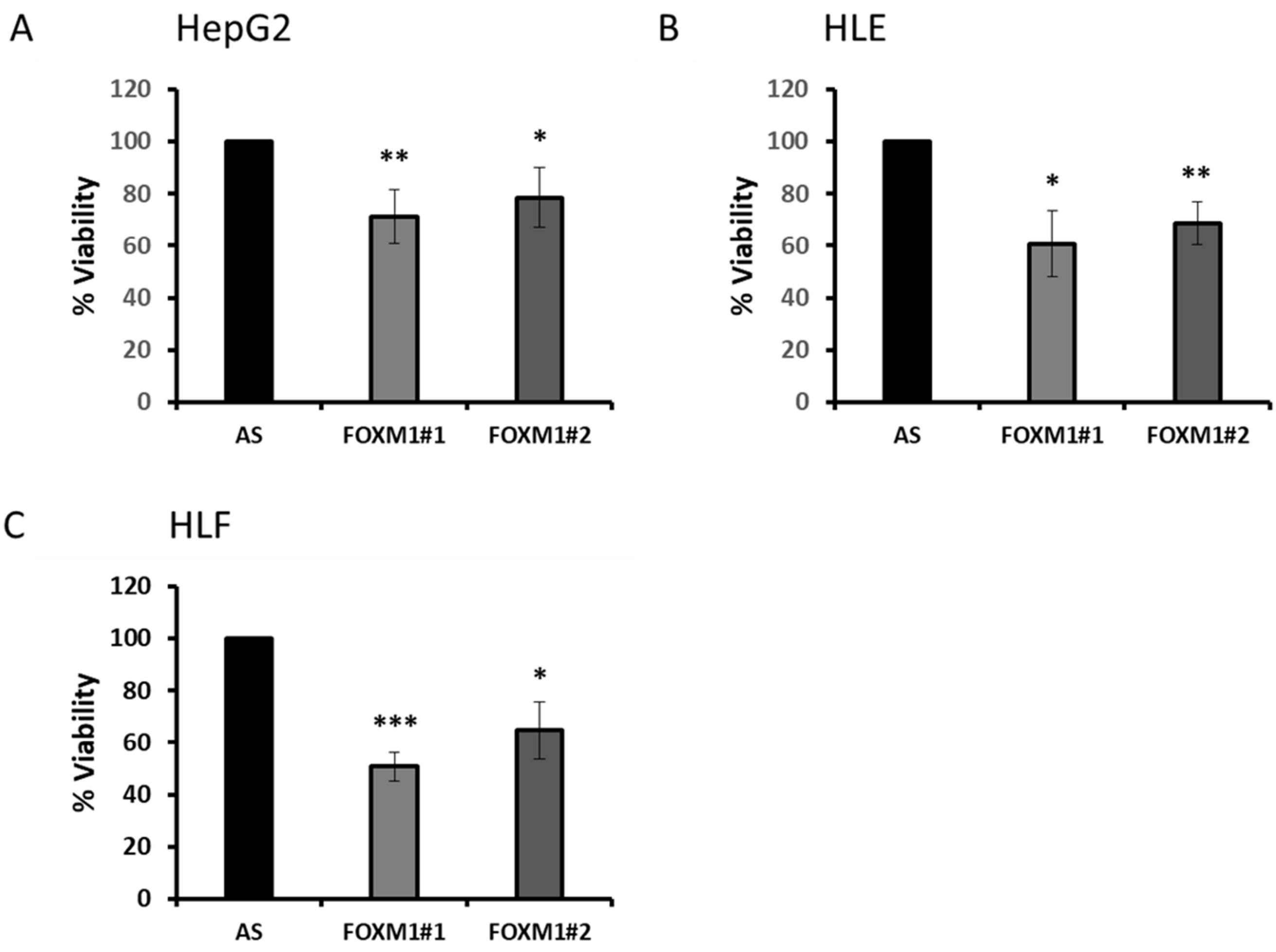

Figure S5. FOXM1 knockdown reduces viability. (A) HepG2 cells were transfected with either control siRNA (AS, Allstars) or two different FOXM1 siRNAs (FOXM1#1 and FOXM1#2). Cell viability was measured by MTT assays 4 days upon FOXM1 knockdown (B) HLE cells were treated as described in (A). (C) HLF cells were treated as described in (A) $*p < 0.05$, $**p < 0.01$, $***p < 0.001$ (Student's t-test). Data are presented as mean ± stdv.

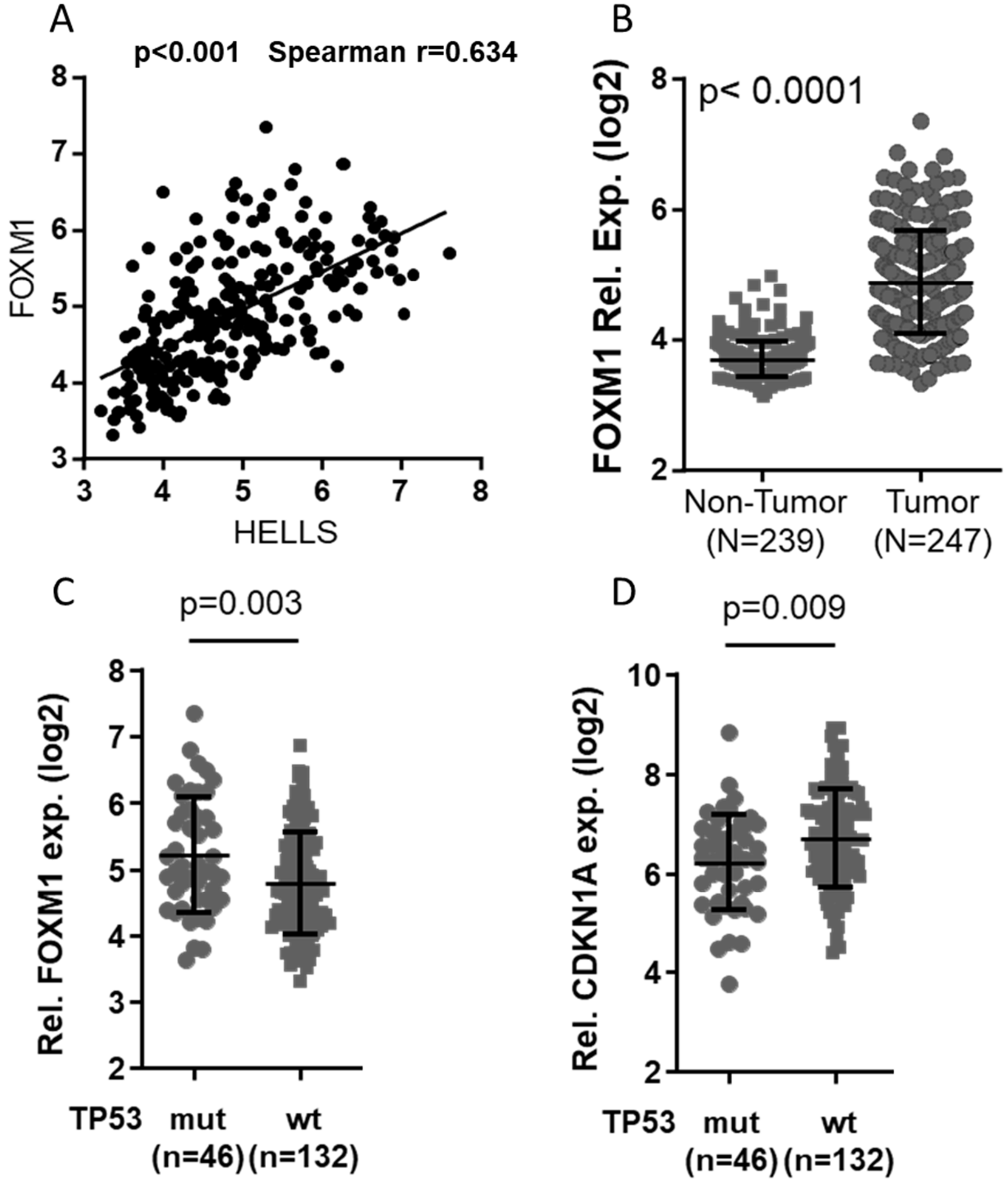

Figure S6. *HELLS, FOXM1* and *CDKN1A* expression levels in human HCC. (A) Correlation between *HELLS* and *FOXM1* expression in human HCC. (B) *FOXM1* expression in Hepatocellular carcinoma (HCC, Tumor) and matching non-tumorous tissue. (C) *FOXM1* expression in HCC harboring either wildtype (wt) or mutated (mut) *TP53*. (D) *CDKN1A* expression in HCC harboring either wildtype (wt) or mutated (mut) *TP53*. Analysis of the data set has been performed as previously published by Roessler et al. [30]. Statistical significance has been determined using the Mann-Whitney test. *p*-values ≤ 0.05 have been considered significant.

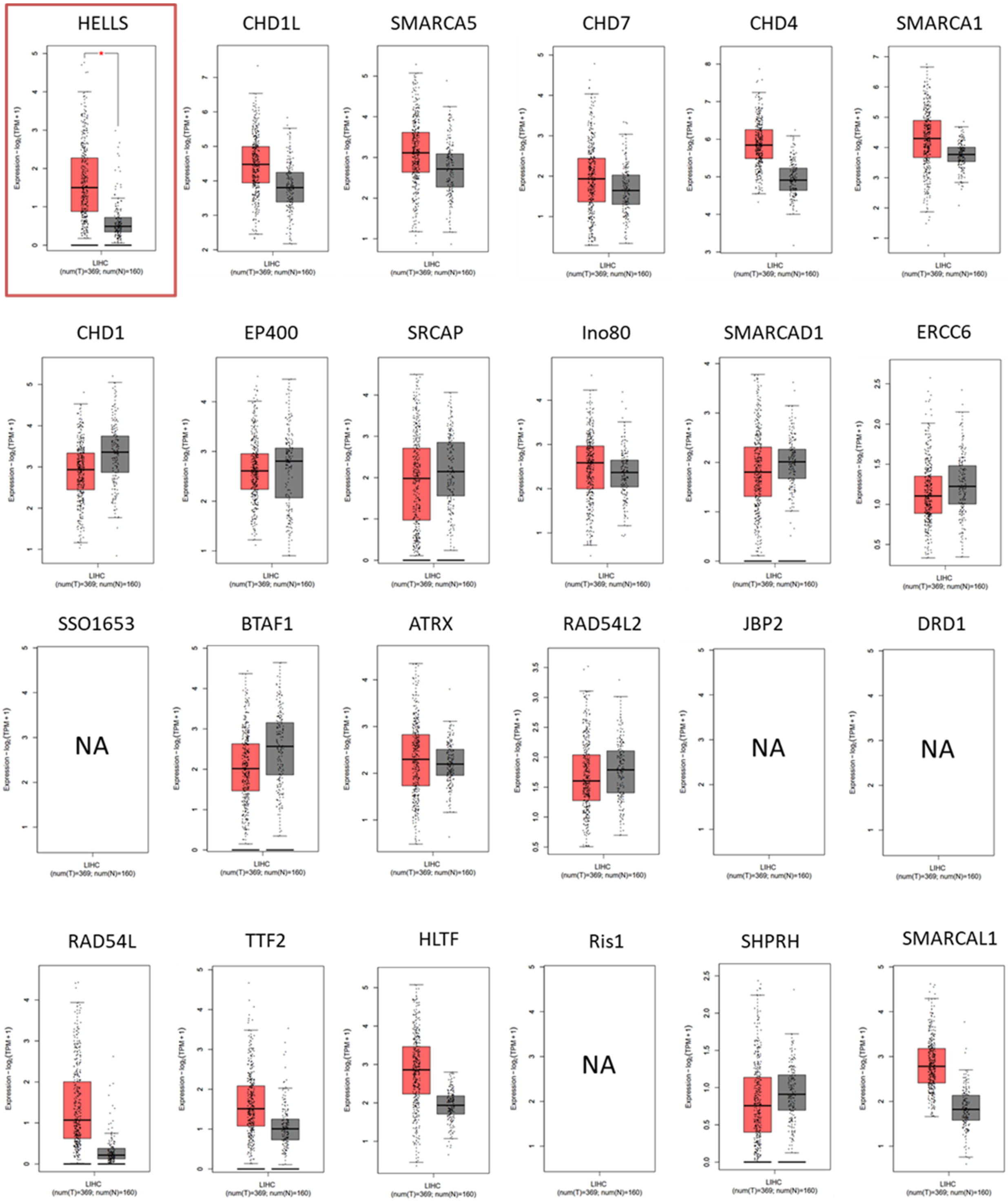

Figure S7. Expression of SNF2 family members in HCC. Comparative expression analysis of SNF2 family members (Flaus et al. [18]) in the TCGA HCC (LIHC) cohort using GEPIA2 [29]. Red boxes represent the expression in tumors (T=369), grey boxes represent non-tumorous tissue (NT=160). Statistically significant (log2 FC cutoff: 1.0; q-value cutoff: 0.01) comparisons are indicated by a red asterisk and frame. NA indicates that this SNF2 family member either has no homologue in humans or that there is no correspondingdata in the TCGA data set.

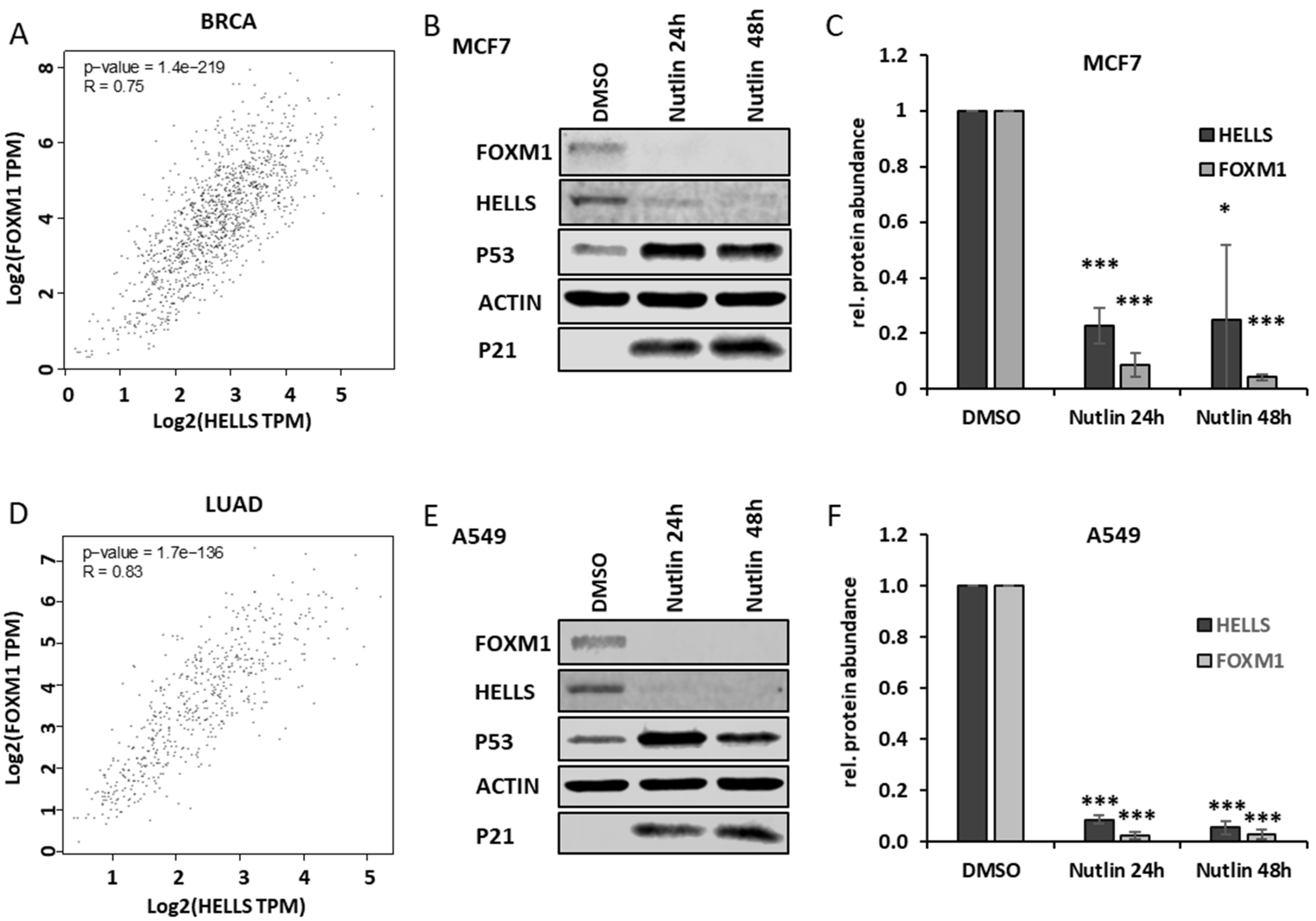

Figure S8. HELLS and FOXM1 expression in human breast cancer and human lung adenocarcinoma. (A) Correlation between *HELLS* and *FOXM1* expression in human breast cancer (BRCA) based on a TCGA data set. (B) MCF7 cells were treated with DMSO or Nutlin-3a for 24h or 48h. Cell extracts were analyzed by immunoblotting with indicated antibodies. (C) Relative densitometric quantification of immunoblots derived from four independent experiments and normalized to the DMSO control as described in (B). (D) Correlation between HELLS and FOXM1 expression in human lung adenocarcinoma (LUAD) based on a TCGA data set. (E) A549 cells were treated with DMSO or Nutlin-3a for 24h or 48h. Cell extracts were analyzed by immunoblotting with indicated antibodies. (F) Relative densitometric quantification of immunoblots derived from four independent experiments and normalized to the DMSO control as described in (E). *$p < 0.05$, **$p < 0.01$, ***$p < 0.001$ (Student’s t-test). Data are presented as mean ± stdv.

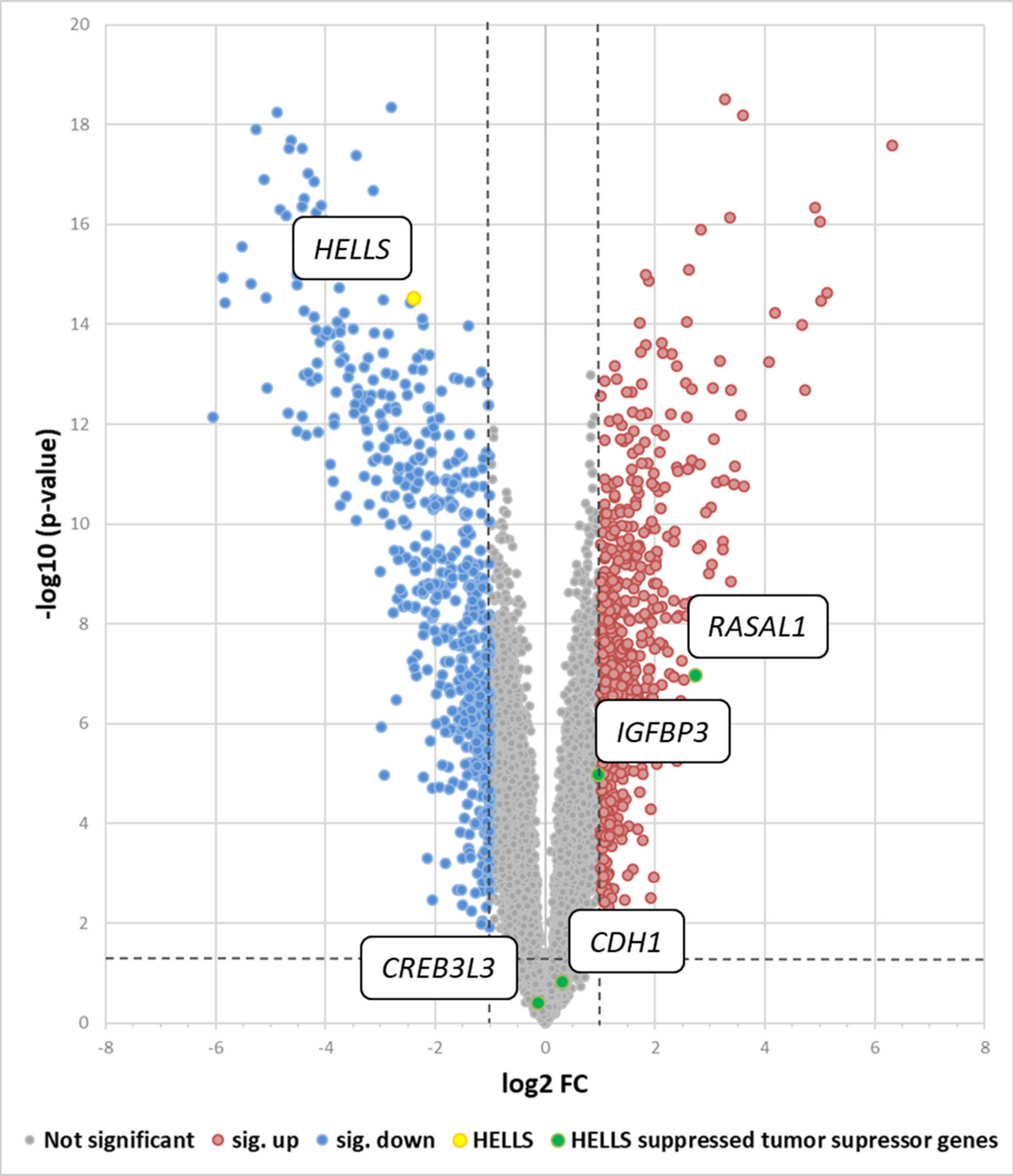

Figure S9. Expression of previously described HELLS-repressed tumor suppressor genes upon Nutlin-3a treatment in HepG2. Volcano plot showing 9129 transcripts identified in HepG2 cells by Affymetrix microarray. 530 transcripts were significantly up- (colored in red) and 571 transcripts were significantly down-regulated (colored in blue) after 24h Nutlin-3a treatment. (Horizontal dotted line p = 0.05; vertical dotted lines log2 fold-change 1.0 or −1.0). mRNA levels of tumor suppressor genes as discussed by Law et al. [19] are highlighted in green.

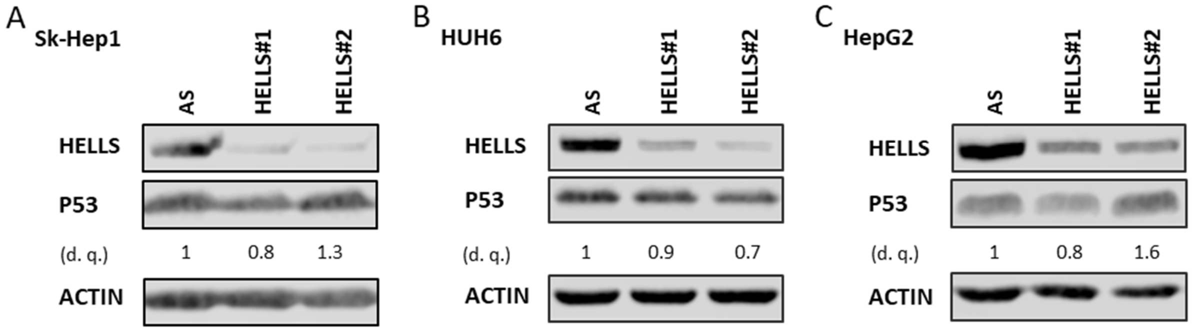

Figure S10. Basal levels of P53 protein are not strikingly and consistently affected by HELLS depletion. (A) Sk-Hep1 cells were transfected with either control siRNA (AS) or two different HELLS siRNAs (HELLS#1 and #2). Cell extracts were analyzed by immunoblotting with indicated antibodies. Densitometric quantification (d. q.) of P53 normalized to control siRNA. (B) HUH6 cells as described in (A). (C) HepG2 cells as described in (A).

Table S1. The 50 strongest up- and downregulated genes after Nutlin-treatment of HepG2 cells.

GeneSymbol	GeneName	log2 FC	Adj. p-value
ABCA12	ATP binding cassette subfamily A member 12	6.31	5.11E-14
SIGLEC14	sialic acid binding Ig like lectin 14	5.14	5.81E-12
LCN15	lipocalin 15	5.02	7.36E-12
TYRP1	tyrosinase related protein 1	5.01	4.28E-13
NBEAP1	neurobeachin pseudogene 1	4.91	2.75E-13
LOC102723373	uncharacterized LOC102723373	4.72	1.80E-10
LCE1F	late cornified envelope 1F	4.67	1.87E-11
DSC3	desmocollin 3	4.17	1.21E-11
PDE4C	phosphodiesterase 4C	4.07	6.89E-11
LINC02334	long intergenic non-protein coding RNA 2334	3.62	6.38E-09
PLTP	phospholipid transfer protein	3.61	2.58E-14
LCE1C	late cornified envelope 1C	3.58	5.12E-07
LOC101927355	uncharacterized LOC101927355	3.57	4.35E-10
IZUMO1R	IZUMO1 receptor, JUNO	3.45	2.94E-09
LOC105373116	uncharacterized LOC105373116	3.43	5.95E-09
S1PR3	sphingosine-1-phosphate receptor 3	3.38	2.50E-07
DRAXIN	dorsal inhibitory axon guidance protein	3.38	1.76E-10
CLCA2	chloride channel accessory 2	3.36	3.82E-13
TP53I3	tumor protein p53 inducible protein 3	3.27	2.58E-14
DENND1C	DENN domain containing 1C	3.25	5.04E-09
OR52N4	olfactory receptor family 52 subfamily N member 4 (gene/pseudogene)	3.24	1.95E-06
TMEM27	transmembrane protein 27	3.24	7.15E-08
LOC101928447	uncharacterized LOC101928447	3.23	5.02E-08
HLA-DOA	major histocompatibility complex, class II, DO alpha	3.17	6.61E-11
CSTA	cystatin A	3.14	6.47E-07
FRMPD2	FERM and PDZ domain containing 2	3.13	5.46E-09
CABYR	calcium binding tyrosine phosphorylation regulated	3.08	1.07E-09
ZNF385A	zinc finger protein 385A	3.06	1.70E-10
PSTPIP2	proline-serine-threonine phosphatase interacting protein 2	3.06	1.67E-06

TREM2	triggering receptor expressed on myeloid cells 2	3.03	1.26E-07
IGFBP1	insulin like growth factor binding protein 1	3.01	1.37E-08
TRIM22	tripartite motif containing 22	2.98	1.82E-07
C1QL3	complement C1q like 3	2.93	1.73E-08
CYGB	cytoglobin	2.84	5.91E-13
LOC105375451	uncharacterized LOC105375451	2.84	6.09E-08
PLEKHG1	pleckstrin homology and RhoGEF domain containing G1	2.81	2.78E-09
CALHM1	calcium homeostasis modulator 1	2.78	6.87E-08
RASAL1	RAS protein activator like 1	2.72	8.26E-06
ANKRA2	ankyrin repeat family A member 2	2.68	1.73E-10
GPRC5B	G protein-coupled receptor class C group 5 member B	2.67	5.23E-07
DPYSL4	dihydropyrimidinase like 4	2.66	2.41E-09
GLS2	glutaminase 2	2.61	2.49E-12
LOC102723561	uncharacterized LOC102723561	2.61	3.23E-09
RRAD	RRAD, Ras related glycolysis inhibitor and calcium channel regulator	2.59	3.38E-09
AMOTL1	angiomotin like 1	2.58	4.67E-10
TNNI2	troponin I2, fast skeletal type	2.58	8.93E-07
LINC02086	long intergenic non-protein coding RNA 2086	2.57	1.71E-11
CEL	carboxyl ester lipase	2.56	1.38E-10
GAL3ST4	galactose-3-O-sulfotransferase 4	2.54	5.69E-07
LINC01173	long intergenic non-protein coding RNA 1173	2.52	9.94E-06

NUF2	NUF2, NDC80 kinetochore complex component	-3.87	5.18E-09
HMGB2	high mobility group box 2	-3.90	2.55E-11
KIF15	kinesin family member 15	-3.91	2.80E-09
TICRR	TOPBP1 interacting checkpoint and replication regulator	-3.97	2.37E-11
BRIP1	BRCA1 interacting protein C-terminal helicase 1	-4.01	2.71E-11
NUSAP1	nucleolar and spindle associated protein 1	-4.01	4.28E-13
MAD2L1	mitotic arrest deficient 2 like 1	-4.06	2.55E-11
KIF14	kinesin family member 14	-4.08	2.72E-13
PLK4	polo like kinase 4	-4.11	3.46E-11
SKA3	spindle and kinetochore associated complex subunit 3	-4.11	8.33E-13
PRC1	protein regulator of cytokinesis 1	-4.14	8.15E-10
CEP55	centrosomal protein 55	-4.15	7.04E-11
HIST1H2AB	histone cluster 1 H2A family member b	-4.15	1.15E-10
KIF18A	kinesin family member 18A	-4.16	3.17E-13
CDC20	cell division cycle 20	-4.16	2.22E-11
GINS2	GINS complex subunit 2	-4.20	1.44E-11
KIF2C	kinesin family member 2C	-4.21	9.77E-13
PLK1	polo like kinase 1	-4.21	1.22E-13
SPAG5	sperm associated antigen 5	-4.23	6.04E-13
SGO1	shugoshin 1	-4.27	1.31E-10
RRM2	ribonucleotide reductase regulatory subunit M2	-4.29	1.81E-12
CCNB2	cyclin B2	-4.31	9.73E-11
SHCBP1	SHC binding and spindle associated 1	-4.32	9.15E-14
FOXM1	forkhead box M1	-4.32	2.69E-12
CENPF	centromere protein F	-4.36	9.26E-10

KIF11	kinesin family member 11	-4.38	1.06E-10
CENPE	centromere protein E	-4.39	2.06E-13
MCM10	minichromosome maintenance 10 replication initiation factor	-4.39	1.12E-11
KIF23	kinesin family member 23	-4.42	4.63E-10
TTK	TTK protein kinase	-4.43	2.72E-13
HMMR	hyaluronan mediated motility receptor	-4.43	5.11E-14
ASPM	abnormal spindle microtubule assembly	-4.50	9.12E-13
KIF20A	kinesin family member 20A	-4.51	4.09E-12
CDK1	cyclin dependent kinase 1	-4.52	8.08E-10
MKI67	marker of proliferation Ki-67	-4.52	2.89E-12
ESCO2	establishment of sister chromatid cohesion N-acetyltransferase 2	-4.63	4.88E-14
PBK	PDZ binding kinase	-4.67	5.11E-14
NCAPH	non-SMC condensin I complex subunit H	-4.68	4.02E-10
CDC6	cell division cycle 6	-4.72	3.69E-13
DLGAP5	DLG associated protein 5	-4.82	3.00E-13
ARHGAP11A	Rho GTPase activating protein 11A	-4.89	2.58E-14
DTL	denticleless E3 ubiquitin protein ligase homolog	-5.06	1.66E-10
HIST1H4D	histone cluster 1 H4 family member d	-5.09	6.80E-12
TOP2A	DNA topoisomerase II alpha	-5.11	1.19E-13
CCNA2	cyclin A2	-5.27	3.66E-14
BUB1	BUB1 mitotic checkpoint serine/threonine kinase	-5.36	3.93E-12
NCAPG	non-SMC condensin I complex subunit G	-5.52	1.10E-12
FAM111B	family with sequence similarity 111 member B	-5.82	8.07E-12
HIST1H3B	histone cluster 1 H3 family member b	-5.87	3.20E-12
HIST1H2BM	histone cluster 1 H2B family member m	-6.04	4.78E-10
